# Metabolic dysfunction-associated steatotic liver disease: pathogenesis and novel treatment options

**DOI:** 10.1186/s43556-026-00486-5

**Published:** 2026-05-30

**Authors:** Ruizhe Ren, Xiao Liang, Xiyang Wei

**Affiliations:** 1 School of Life Sciences, Zhejiang Chinese Medical University, Hangzhou, Zhejiang 310053 China; 2 Zhejiang Key Laboratory of Multi-Omics Precision Diagnosis and Treatment of Liver Diseases, Department of General Surgery, Sir Run-Run Shaw Hospital, Zhejiang University School of Medicine, Hangzhou, 310016 China; 3 School of Medicine, Shaoxing University, Shaoxing, 312000 China; 4 School of Basic Medical Sciences and Forensic Medicine, Hangzhou Medical College, Hangzhou, 310000 China

**Keywords:** MASLD, MASH, Pathogenesis, Diagnosis, Novel therapeutics

## Abstract

Metabolic dysfunction-associated steatotic liver disease (MASLD), the recently introduced terminology superseding non-alcoholic fatty liver disease (NAFLD), has emerged as the most prevalent chronic liver condition globally. This multisystem disorder, driven by systemic insulin resistance (IR) and metabolic dysregulation, not only progresses to cirrhosis and hepatocellular carcinoma (HCC) but also substantially increases the risk of cardiovascular disease (CVD), type 2 diabetes (T2D), and extrahepatic malignancies. Despite the recent FDA approval of resmetirom for a subset of patients with advanced fibrosis, effective pharmacological therapies for the broad MASLD population remain a critical unmet need, underscoring the urgency of continued research into disease mechanisms and therapeutic targets. This review synthesizes recent advances in three interconnected domains: the complex pathogenesis of MASLD (including lipotoxicity, mitochondrial dysfunction, inflammation, and fibrogenesis), the evolving landscape of non-invasive diagnostic tools, and the development of novel therapeutic agents targeting key pathways. By critically analyzing both successful late-stage trials and instructive failures, we highlight the challenges of translating mechanistic insights into clinically meaningful outcomes. By providing a comprehensive, integrated overview of current knowledge and future directions, this review aims to serve as a valuable resource for researchers and clinicians working toward effective, personalized interventions for this global health challenge.

## Introduction

**Fig. 1:**
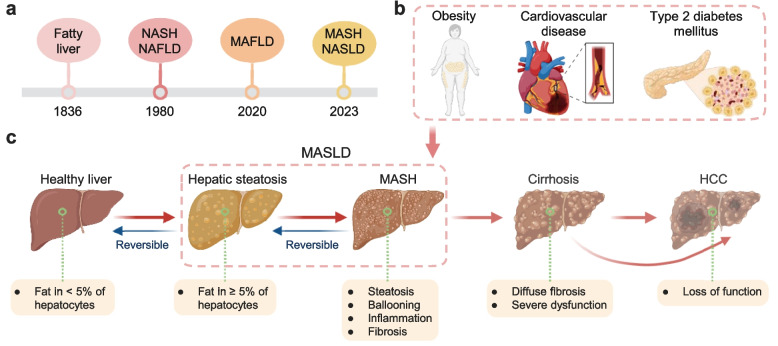
Overview of MASLD. **a** Timeline of nomenclature evolution. The conceptual framework for fatty liver disease has evolved from its initial description in 1836 to the current consensus terminology. **b** MASLD as a multisystem metabolic disorder. MASLD is closely intertwined with key components of the metabolic syndrome including obesity, type 2 diabetes, and cardiovascular disease. **c** Pathological spectrum of MASLD progression. The disease progresses along a histological continuum from normal liver to isolated hepatic steatosis, followed by the development of MASH. Persistent injury leads to cirrhosis and, in a subset of patients, hepatocellular carcinoma. Abbreviations: NASH, nonalcoholic steatohepatitis; NAFLD, nonalcoholic fatty liver disease; MAFLD, metabolic dysfunction-associated fatty liver disease; MASLD, metabolic dysfunction-associated steatotic liver disease; MASH, metabolic dysfunction-associated steatohepatitis. Created in BioRender. Wei, X. (2026) https://BioRender.com/dpnvtbh

The nomenclature of fatty liver disease has evolved markedly since its first description by Addison in 1836, culminating in the 1980 introduction of “nonalcoholic steatohepatitis” (NASH) and the subsequent establishment of non-alcoholic fatty liver disease (NAFLD) as an overarching concept [1–3]. This historical framework, however, defined the disease by exclusion, which requires the absence of significant alcohol consumption, rather than by its underlying biology [4]. A pivotal shift occurred in 2020 when Eslam and colleagues proposed “metabolic dysfunction-associated fatty liver disease” (MAFLD), diagnosed by hepatic steatosis together with overweight/obesity, type 2 diabetes (T2D), or at least two metabolic risk abnormalities [5, 6]. Most recently, a 2023 multinational consensus recommended updating the terminology to “metabolic dysfunction-associated steatotic liver disease” (MASLD) and “metabolic dysfunction-associated steatohepatitis” (MASH), further refining the diagnostic framework to affirmatively capture metabolic etiology [7] (Fig. 1a).

This evolution from a “non-alcoholic” diagnosis of exclusion to an affirmative “metabolic dysfunction”-based framework represents more than terminological revision—it fundamentally reorients our understanding of the disease. By replacing exclusionary criteria with positive metabolic definitions, the MASLD framework reduces stigmatization, encourages proactive screening for cardiometabolic risk factors, and aligns clinical practice with the systemic nature of the condition [8]. However, important tensions persist. The MAFLD and MASLD criteria, while conceptually aligned, differ in their diagnostic thresholds and metabolic requirements: MAFLD offers broader inclusion through its three-category approach, whereas MASLD adopts a more stringent five-factor cardiometabolic model. This discrepancy creates transitional challenges for interpreting historical literature and designing clinical trials, and ongoing debates regarding optimal cut-off values for metabolic parameters underscore the need for continued refinement [9]. Furthermore, while the new framework appropriately captures most patients, “lean MASLD”, which affects normal-weight individuals with metabolic dysfunction, remains a diagnostically challenging subset. Although these individuals now fall within the MASLD diagnostic framework by meeting at least one cardiometabolic criterion, their clinical identification is often delayed due to the absence of overt obesity, and the underlying disease mechanisms in this population remain incompletely understood [10]. Thus, the controversy has shifted from diagnostic inclusion toward the need for earlier recognition strategies and a deeper understanding of distinct pathophysiological drivers in lean versus obese MASLD phenotypes.

MASLD has emerged as the most prevalent chronic liver disease worldwide, currently affecting approximately 30% to 40% of the global adult population, and 7% to 14% of children and adolescents (pediatric MASLD) [11, 12]. Between 1990 and 2021, the number of affected individuals more than doubled, reaching 1.3 billion, and model-based forecasts project that global prevalence will surpass 55% by 2040 [13, 14]. This alarming trajectory is particularly pronounced in China, which reports among the highest incidence rates globally, driven by dietary shifts toward ultra-processed foods, increasingly sedentary lifestyles, and an aging population [15–17]. The rising burden of MASLD mirrors the epidemics of obesity and T2D: meta-analyses reveal MASLD prevalence of 70% and 75% in overweight and obese populations, respectively, and approximately 68.8% among individuals with T2D [18, 19]. Beyond its hepatic consequences, MASLD is independently associated with increased cardiovascular disease (CVD), the leading cause of mortality in patients without advanced fibrosis (Fig. 1b). However, once cirrhosis develops, liver-related mortality, including deaths from complications of cirrhosis and hepatocellular carcinoma (HCC), rises substantially [20, 21]. MASLD is also associated with elevated risks of multiple extrahepatic malignancies, including colorectal, pancreatic, breast, and thyroid cancers [22].

MASLD encompasses a dynamic clinicopathological spectrum (Fig. 1c), ranging from isolated hepatic steatosis to MASH (defined by lobular inflammation and hepatocyte ballooning), and ultimately progressive fibrosis that determines long-term clinical outcomes [21]. It is estimated that approximately 30% of individuals with MASLD will progress to MASH, a transition that critically accelerates the process of fibrogenesis [23]. The subsequent development and progression of liver fibrosis, systematically staged from F0 (no fibrosis) to F4 (compensated or decompensated cirrhosis), serves as the single most critical predictor of long-term clinical outcomes, effectively bridging hepatic injury with hard endpoints [24]. While CVD consistently remains the leading cause of mortality across the entire MASLD spectrum, the risk of liver-related morbidity and mortality increases exponentially with the fibrosis stage, with the vast majority of these severe events occurring in patients with advanced (F3-F4) fibrosis [25]. While early-stage disease is often reversible through substantial weight loss and metabolic management, advanced cirrhosis carries a grim prognosis and may necessitate liver transplantation, underscoring the urgency of identifying and treating “at-risk MASH” [26, 27].

Despite the substantial and escalating global burden of MASLD and the recent FDA approval of resmetirom for a subset of patients with advanced fibrosis, effective pharmacological therapies for the broad MASLD population remain a critical unmet need, and the field continues to grapple with fundamental questions regarding optimal diagnostic strategies, patient stratification, and therapeutic targeting [28]. This review synthesizes recent advances in three interconnected domains: the complex pathogenesis of MASLD, the evolving landscape of diagnostic approaches, and the development of novel therapeutic agents targeting key pathway. By critically analyzing both successful late-stage trials and instructive failures, we aim to provide a comprehensive, integrated resource that informs future research directions and accelerates the development of effective, personalized interventions for this increasingly prevalent disease.

## The complex pathogenic network of MASLD

### Metabolic dysregulation and mitochondrial dysfunction

**Fig. 2:**
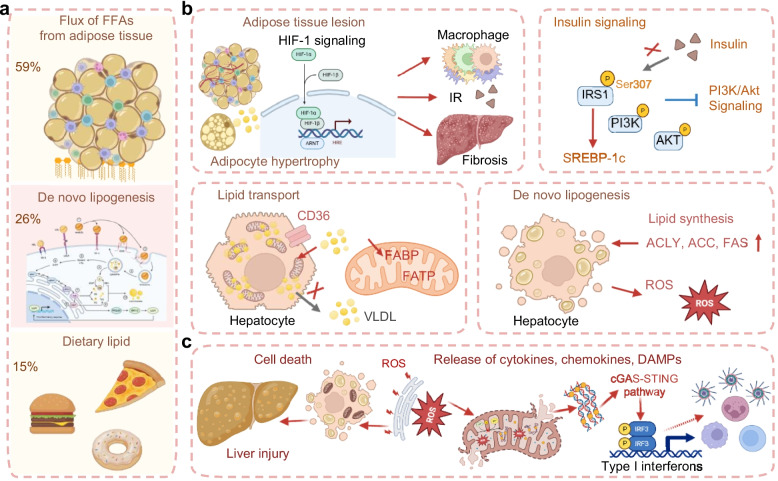
Central role of metabolic dysregulation and mitochondrial dysfunction in MASLD pathogenesis. **a** Principal sources of hepatic lipid accumulation. Hepatic steatosis, the hallmark of MASLD, is driven by three major lipid sources: increased flux of FFAs from adipose tissue, enhanced hepatic DNL, and direct dietary lipid uptake. **b** Mechanistic links between metabolic drivers and hepatic injury. Adipose tissue IR promotes excessive FFA release into the circulation, which, together with hyperinsulinemia, activates the lipogenic transcription factor SREBP-1c, thereby driving sustained DNL. **c** Mitochondrial dysfunction as a key amplifier of disease progression. In the lipotoxic environment, mitochondrial function becomes compromised. ROS triggers oxidative damage, promote the release of cytokines, chemokines and DAMPs, thereby activating pro-inflammatory signaling cascades (e.g., cGAS-STING). Abbreviations: FFAs, free fatty acids; DNL, de novo lipogenesis; IR, insulin resistance; ROS, reactive oxygen species; VLDL, very-low-density lipoproteins; ACLY, ATP-citrate lyase; ACC, acetyl-CoA carboxylase; FAS, fatty acid synthase; DAMPs, damage-associated molecular patterns. Created in BioRender. Wei, X. (2026) https://BioRender.com/5s0kihv

Increased flux of free fatty acids (FFAs) from adipose tissue driven by insulin resistance (IR), enhanced hepatic de novo lipogenesis (DNL) stimulated by carbohydrates and insulin, and dietary lipid uptake are main drivers of excessive hepatic lipid accumulation (steatosis), which is the hallmark pathological feature of MASLD [27]. Quantitative studies have established that these three sources account for approximately 59%, 26%, and 15% of hepatic triglycerides, respectively [29] (Fig. 2a). This pathologic lipid accumulation, particularly when compounded by defective lipid handling, sets the stage for a deleterious cascade. Lipotoxicity emerges as the central event, triggering mitochondrial dysfunction and oxidative stress, which in turn ignite a chronic inflammatory response. The resulting self-perpetuating cycle of metabolic insult and inflammation ultimately drives the key pathological outcomes of MASLD: direct hepatocyte injury, progressive fibrogenesis, and an elevated risk of carcinogenesis [30].

During the development of obesity, adipose tissue undergoes profound metabolic alterations that contribute significantly to the pathogenesis of MASLD. Adipose tissue expansion becomes pathological, characterized by adipocyte hypertrophy, limited angiogenesis, and localized hypoxia. These conditions activate HIF-1 signaling, promote fibrosis, and induce systemic IR, accompanied by proinflammatory macrophage infiltration [31] (Fig. 2b). As insulin’s normal action is to suppress lipolysis, the onset of adipose IR pathologically unleashes an excessive release of FFAs into the circulation—a hallmark feature consistently observed in individuals with MASLD [32]. The ensuing flux of FFAs to the liver instigates lipotoxicity, promoting the accumulation of harmful lipid species that trigger hepatic metabolic stress, inflammation, and the progression of liver disease [33].

The canonical insulin signaling cascade initiates with insulin binding to its receptor, leading to tyrosine phosphorylation of insulin receptor substrates (IRS1 and IRS2), which in turn recruits and activates the PI3K/Akt pathway to mediate metabolic responses (Fig. 2b). In the lipotoxic milieu characteristic of MASLD, however, this signaling axis becomes profoundly compromised. A pivotal mechanism underlying this dysfunction involves the aberrant serine/threonine phosphorylation of IRS proteins such as phosphorylation at Ser307 in IRS1 [34, 35]. This inhibitory modification is catalyzed by multiple stress-activated kinases upregulated in MASLD, including JNK1 (activated by ER stress and FFA), IKKβ (connecting inflammatory signaling to IR), mTORC1 (stimulated by nutrient and amino acid surplus), and specific PKC isoforms (activated by lipid metabolites such as diacylglycerols). Serine-phosphorylated IRS proteins undergo functional inactivation, enhanced degradation, or impaired docking with the insulin receptor, collectively blunting downstream insulin signal transduction [36, 37]. This blockade not only abrogates insulin-mediated suppression of hepatic glucose output but also establishes a metabolic paradox: although anabolic insulin signaling through the PI3K/Akt axis is impaired, compensatory hyperinsulinemia persistently activates the lipogenic transcription factor SREBP-1c. This sustained SREBP-1c activation drives uninterrupted DNL, thereby further aggravating hepatic lipid accumulation and creating a self-reinforcing cycle of steatosis and metabolic dysfunction [32, 38].

In both MASLD and MASH, DNL is markedly upregulated and closely associated with hepatic IR [39]. This pathway converts substrates including glucose, fructose, lactate, acetate, and amino acids into fatty acids, primarily under the transcriptional control of SREBP-1c (activated by insulin signaling) and ChREBP (stimulated by glucose) [40, 41]. Together, these regulators upregulate key DNL enzymes including ATP-citrate lyase (ACLY), acetyl-CoA carboxylase (ACC), and fatty acid synthase (FAS), creating a “double hit” that accelerates lipid synthesis [42] (Fig. 2b). In response to lipid overload, compensatory elevations in fatty acid oxidation and TCA cycle activity occur. However, under chronic conditions, this response ultimately intensifies oxidative stress and aggravates hepatocyte injury [43].

Beyond enhanced DNL and disrupted fatty acid oxidation, MASLD involves a pathological upregulation of key fatty acid transport proteins, including fatty acid-binding protein (FABP), fatty acid transport protein (FATP), and the scavenger receptor CD36 (Fig. 2b). This increased expression on the hepatocyte membrane significantly facilitates the uncontrolled uptake of circulating fatty acids, further exacerbating hepatic lipid burden [44–46]. Concurrently defects in the packaging and export of triglycerides via very-low-density lipoproteins (VLDL) constitute a major contributor to disease progression [32]. Crucially, an early key impairment is the liver’s failure to respond to normal insulin signaling that should suppress VLDL secretion. This loss of insulin-mediated suppression creates a pathogenic state of uncontrolled lipid release amidst ongoing lipid influx, profoundly exacerbating hepatic steatosis and highlighting the profound metabolic dysregulation in MASLD [47].

A critical defect observed in MASLD progression is mitochondrial inefficiency and dysfunction (Fig. 2c). The heightened flux of electrons through the mitochondrial electron transport chain (ETC), coupled with lipid-derived toxic metabolites (e.g., dicarboxylic acids), promotes a state of oxidative stress [48]. Specifically, mitochondrial uncoupling, a process where protons leak back into the mitochondrial matrix without generating ATP, becomes prominent. This can be mediated by the upregulation of uncoupling proteins (UCPs) or by the permeability transition pore [49]. While mild uncoupling can theoretically reduce ROS production, in the context of MASLD, it is often excessive and dysregulated, leading to a futile cycle of substrate oxidation that compromises energy (ATP) synthesis and further increases electron leak from complexes I and III. Consequently, reactive oxygen species (ROS), particularly superoxide anions (O₂•⁻) and hydrogen peroxide (H₂O₂), are generated in excess, overwhelming the antioxidant defense systems (e.g., glutathione, superoxide dismutase) [50].

The consequences of this mitochondrial dysfunction are twofold. First, the elevated ROS directly damage cellular macromolecules, including mitochondrial DNA (mtDNA), lipids (initiating lipid peroxidation chains), and proteins, triggering hepatocyte apoptosis and necroptosis. Second, ROS act as potent signaling molecules that activate multiple pro-inflammatory pathways [51]. They can directly activate the NLRP3 inflammasome within Kupffer cells and hepatocytes, leading to the maturation and secretion of interleukin-1β (IL-1β) and IL-18. Furthermore, ROS oxidize mtDNA, which, once released into the cytosol, can act as damage-associated molecular patterns (DAMPs) to engage innate immune sensors like cGAS-STING and TLR9, thereby amplifying the production of type I interferons and other inflammatory cytokines [52–54]. This creates a vicious cycle where metabolic stress induces mitochondrial ROS, which in turn fuels hepatic inflammation and injury, driving disease progression from simple steatosis to MASH. Notably, genes involved in fatty acid oxidation, such as PPARγ and CPT1, are upregulated, whereas PGC-1α, a central regulator of mitochondrial biogenesis, is downregulated [48, 55, 56].

In summary, MASLD pathogenesis originates from disrupted hepatic lipid homeostasis. Excessive lipid influx, which is driven by adipose dysfunction, insulin resistance, enhanced de novo lipogenesis, and impaired lipid export, establishes chronic lipotoxicity. This lipotoxic milieu directly compromises mitochondrial integrity and function. The resulting mitochondrial inefficiency, marked by respiratory chain uncoupling and excessive ROS production, transforms metabolic overload into sustained oxidative stress and inflammatory signaling. Mitochondrial dysfunction thus acts as a critical amplifier, converting initial metabolic disturbance into progressive hepatocyte injury and fibrogenesis. Strategically, this hierarchical understanding positions key regulators of lipid metabolism (such as SREBP-1c, ChREBP, and CD36) and mitochondrial quality control pathways (including PGC-1α and mitophagy regulators) as compelling therapeutic targets. Intervening at either the lipotoxic source or its mitochondrial consequences, or ideally both, represents a rational strategy to disrupt the self-perpetuating cycle that drives MASLD progression.

### Activation of inflammation and fibrosis progression

**Fig. 3:**
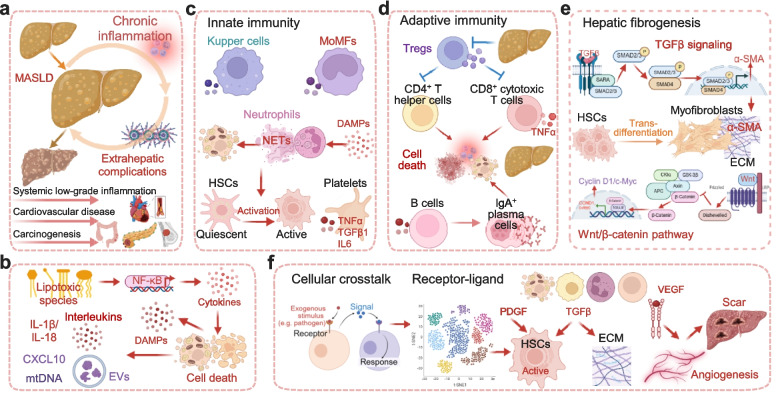
The inflammatory and fibrotic cascade in MASLD progression. **a** Chronic hepatic inflammation as a central driver. Persistent, low-grade systemic inflammation serves as a critical bridge linking simple steatosis to advanced fibrosis and extrahepatic complications, including cardiovascular disease and carcinogenesis. **b** Multiple triggers of the inflammatory cascade. The inflammatory response is initiated and sustained by diverse stimuli. Lipotoxic species act as potent instigators by upregulating proinflammatory cytokines and directly activating the NF-κB signaling pathway. Injured hepatocytes amplify the response by releasing DAMPs, secreting interleukins (IL-1β, IL-18) via inflammasome activation, and dispatching EVs carrying inflammatory mediators (e.g., CXCL10, mtDNA) to neighboring cells. **c** Role of innate immunity. Hepatic macrophages, including resident Kupffer cells and recruited MoMFs, serve as central executors of inflammation. Neutrophils contribute through the formation of NETs, which promote hepatocyte death and activate HSCs. Platelets further amplify inflammation by releasing pro-inflammatory mediators. **d** Role of adaptive immunity. Dysfunction of Tregs leads to loss of immune tolerance, enabling uncontrolled activation of autoreactive CD4⁺ and CD8⁺ T cells. B cells contribute by producing autoantibodies to perpetuate inflammation and fibrosis. **e** Mechanisms of hepatic fibrogenesis. Activated HSCs undergo transdifferentiation into proliferative myofibroblasts, which secrete excessive ECM components. The TGF-β/Smad signaling pathway is the master driver of HSC activation, while the Wnt/β-catenin pathway promotes HSC proliferation and survival. These pathways synergize to sustain the fibrotic response. **f** Cellular crosstalk in advanced fibrosis. Single-nucleus RNA sequencing has revealed conserved receptor-ligand interactions between cell populations in human and murine MASH livers, defining a communication network that sustains HSC activation in advanced fibrosis. Key soluble factors shape this pro-fibrogenic microenvironment: TGF-β, PDGF, and VEGF. Together, they create a self-reinforcing cycle of scar tissue deposition. Abbreviations: DAMPs, damage-associated molecular patterns; NETs, neutrophil extracellular traps; MoMFs, monocyte-derived macrophages; HSCs, hepatic stellate cells; Tregs, regulatory T cells; ECM, extracellular matrix. Created in BioRender. Wei, X. (2026) https://BioRender.com/nv4f2up

Chronic hepatic inflammation is a pivotal driver in the progression of MASLD, critically bridging simple steatosis to advanced liver fibrosis and a spectrum of extrahepatic complications [57]. This hepatic inflammation exists within a context of persistent, systemic low-grade inflammation, a hallmark of MASLD and related metabolic disorders that is independently recognized for elevating the risk of CVD and carcinogenesis [58, 59] (Fig. 3a).

The inflammatory cascade is initiated and sustained by multiple triggers (Fig. 3b). A diverse array of lipotoxic species, including sphingolipids, ceramides, trans fatty acids, and free cholesterol, acts as potent instigators by upregulating proinflammatory cytokines and directly activating the central NF-κB signaling pathway. Furthermore, a growing repertoire of less-characterized lipid mediators is suspected to exacerbate this lipotoxicity-related inflammation [60, 61]. These processes create a vicious cycle by further aggravating hepatic IR, with NF-κB activation playing a particularly crucial role in disrupting insulin signaling [62, 63]. Subsequently, injured hepatocytes amplify the inflammatory response through multiple mechanisms: they release DAMPs such as ATP and histones that activate pattern recognition receptors (PRRs) on innate immune cells; they secrete potent interleukins (IL-1β, IL-18) via inflammasome activation; and they dispatch extracellular vesicles (EVs) carrying inflammatory mediators like CXCL10 and mtDNA, which propagate signals to neighboring cells [64–66].

Innate immunity, particularly the responses mediated by hepatic macrophages, serves as the central executor in perpetuating this inflammatory milieu [67] (Fig. 3c). The hepatic macrophage population, comprising resident Kupffer cells and recruited monocyte-derived macrophages (MoMFs), exhibits remarkable plasticity and functional diversity. These cells are key contributors to inflammation, tissue injury, and fibrogenesis, yet certain subsets also possess the capacity to participate in inflammation resolution and fibrosis regression, highlighting their complex dual role in MASLD pathogenesis [68]. Beyond macrophages, neutrophils and their released structures, known as neutrophil extracellular traps (NETs), contribute to acute inflammatory bursts and tissue injury in MASH. NETs are web-like structures composed of decondensed chromatin, histones, and granular enzymes like myeloperoxidase (MPO) and neutrophil elastase. They are extruded by neutrophils in a process called netosis, originally intended to trap and neutralize pathogens [69]. However, in the metabolic context of MASH, DAMPs and pro-inflammatory cytokines can trigger excessive NET formation. These NETs then directly induce hepatocyte death and activate hepatic stellate cells (HSCs), thereby amplifying inflammation and directly promoting fibrogenesis [70]. Platelets further augment inflammation by infiltrating injured livers and releasing a spectrum of inflammatory and profibrogenic mediators such as TNFα, IL-6, transforming growth factor-beta (TGF-β), various growth factors, and cytokine-loaded microparticles [71–73].

The adaptive immune system also plays a significant, albeit less characterized role in MASLD progression (Fig. 3d). A key mechanism involves the dysfunction of regulatory T cells (Tregs), which are crucial for maintaining peripheral immune tolerance. In a healthy liver, Tregs suppress the activation and effector functions of pro-inflammatory CD4^+^ T helper cells and CD8^+^ cytotoxic T cells. In the MASH microenvironment, however, Treg abundance and suppressive function are often impaired. This loss of immune tolerance allows for the uncontrolled activation of autoreactive T cells that target stressed or injured hepatocytes, creating a feed-forward loop of inflammation and cell death [74, 75]. Furthermore, B lymphocytes contribute to disease pathogenesis through the production of autoantibodies. In response to chronic exposure to neo-antigens released from damaged hepatocytes, B cells can become activated and differentiate into antibody-producing plasma cells. These cells generate immunoglobulins against various self-antigens, such as lipid peroxidation-derived adducts or mitochondrial components. The resulting immune complexes can deposit in the liver, further activating Kupffer cells and the complement system, which in turn exacerbates inflammation and drives fibrosis progression [76, 77].

Hepatic fibrogenesis, the hallmark of progressive MASLD, is primarily driven by the activation of HSCs (Fig. 3e). In response to a complex milieu of inflammatory and metabolic insults, quiescent, vitamin A-storing HSCs undergo a dramatic phenotypical transformation into highly proliferative and contractile myofibroblasts [78, 79]. This activated state is characterized by elevated expression of alpha-smooth muscle actin (α-SMA), massive production of fibrillar collagen type I and other extracellular matrix (ECM) components, and the secretion of a wide array of pro-fibrotic and pro-inflammatory mediators that further perpetuate the disease process [80]. The activation stimuli are diverse, encompassing paracrine signals from activated macrophages, DAMPs released from injured hepatocytes, and a battery of cytokines (e.g., PDGF, TNF-α) from inflamed immune cells [81]. Among these, the TGF-β signaling pathway is the most potent driver of HSC activation. Upon binding to its receptors, TGF-β activates downstream Smad proteins (primarily Smad2 and Smad3). Phosphorylated Smad2/3 forms a complex with Smad4, which then translocate to the nucleus to regulate the transcription of profibrotic genes, including those encoding collagen type I alpha 1 chain (COL1A1) and α-SMA. The TGF-β/Smad axis is thus instrumental not only in initiating the activation of quiescent HSCs but also in robustly sustaining the profibrotic, matrix-producing phenotype of myofibroblasts, making it a master regulator of liver fibrosis [82]. Furthermore, the Wnt/β-catenin pathway plays a critical and complementary role. In the canonical pathway, binding of Wnt ligands to Frizzled receptors stabilizes β-catenin, allowing it to accumulate and translocate into the nucleus. There, it partners with T-cell factor/lymphoid enhancer factor (TCF/LEF) transcription factors to promote the expression of target genes such as Cyclin D1 and c-Myc, which drive HSC proliferation and survival. Notably, sustained Wnt/β-catenin signaling interacts with the TGF-β pathway, creating a synergistic loop that reinforces HSC activation and inhibits apoptosis, thereby perpetuating the fibrotic response [83].

The complexity of this cellular crosstalk is further illuminated by recent single-nucleus RNA sequencing studies, which have identified conserved receptor-ligand interactions between specific cell populations in both human and murine MASH livers, revealing a precise communication network that sustains HSC activation particularly in advanced fibrosis [84] (Fig. 3f). This pro-fibrogenic microenvironment is shaped by key soluble factors: TGF-β, produced by immune and damaged cells, stands as a master inducer of HSC activation and ECM production, while other factors like Platelet-Derived Growth Factor (PDGF) potently drive HSC proliferation and Vascular Endothelial Growth Factor (VEGF) supports fibrogenesis by promoting angiogenesis, together creating a self-reinforcing cycle of scar tissue deposition [68, 85].

In summary, MASLD progression is fueled by a vicious cycle of inflammation and fibrosis. In this cycle, metabolic stress and hepatocyte injury initiate inflammatory responses, which in turn activate fibrogenic mechanisms, ultimately leading to liver dysfunction and associated systemic complications. This intricate interplay underscores that disrupting the crosstalk between inflammatory cells and activated HSCs—for instance, by targeting shared signaling nodes such as TGF-β or inflammasome-derived cytokines—may offer greater therapeutic leverage than intervening in either process alone. Elucidating the temporal dynamics of this cellular dialogue will be critical for designing interventions that halt, and potentially reverse, fibrosis progression.

### Genetic predisposition and epigenetic regulation

**Fig. 4:**
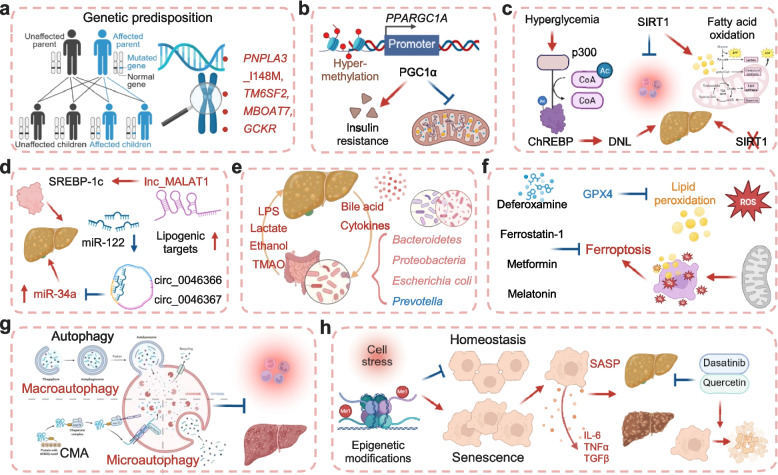
Multilayered regulatory networks in MASLD pathogenesis. **a** Genetic predisposition. Genome-wide association studies have identified several common genetic variants robustly associated with MASLD susceptibility and progression, including *PNPLA3* I148M, *TM6SF2*, *MBOAT7*, and *GCKR*. **b** Epigenetic regulation via DNA methylation. Hypermethylation of the *PPARGC1A* promoter, which encodes the master mitochondrial biogenesis regulator PGC-1α, suppresses its expression and is associated with impaired mitochondrial function and insulin resistance in MASLD patients. **c** Histone modifications. The NAD⁺-dependent deacetylase SIRT1 exerts protective effects by promoting fatty acid oxidation and mitigating inflammation, whereas hyperglycemia-induced activation of the acetyltransferase p300 promotes DNL through acetylation and activation of the transcription factor ChREBP. **d** Non-coding RNA networks. A vast repertoire of non-coding RNAs contributes to epigenetic dysregulation. Downregulation of the liver-abundant miR-122 derepresses lipogenic targets, while elevated miR-34a correlates with disease severity. Competing endogenous RNAs, such as circular RNAs that sequester miR-34a, and long non-coding RNAs like MALAT1 further modulate inflammatory and metabolic pathways. **e** Gut-liver axis and microbial dysbiosis. Dysbiosis compromises intestinal barrier integrity, leading to translocation of microbial products into the portal circulation. For example, LPS triggers pro-inflammatory cascades and hepatic insulin resistance. Gut microbes also modulate bile acids signaling, and produce metabolites such as lactate, ethanol, and TMAO. **f** Ferroptosis. Ferroptosis is characterized by mitochondrial shrinkage, with GPX4 serving as its central gatekeeper; GPX4 upregulation ameliorates metabolic injury. Therapeutic strategies targeting ferroptosis in MASLD focus on inhibiting iron accumulation and lipid peroxidation. Preclinically, iron chelators (e.g., deferoxamine), radical-trapping agents (e.g., ferrostatin-1), and compounds such as metformin and melatonin attenuate hepatocyte injury, inflammation, and fibrosis. **g** Autophagy. Autophagy encompasses several forms, including macroautophagy, CMA, microautophagy, and crinophagy. **h** Cellular senescence. In MASLD, epigenetic modifications drive cellular senescence. Senescent cells secrete pro-inflammatory and pro-fibrotic factors (SASP), perpetuating tissue damage. Senolytics (e.g., dasatinib + quercetin) selectively clear these cells, ameliorating liver inflammation and fibrosis in preclinical models, with early-phase clinical trials ongoing. Abbreviations: DNL, de novo lipogenesis; LPS, lipopolysaccharide; TMAO, trimethylamine N-oxide; CMA, chaperone-mediated autophagy; SASP, senescence-associated secretory. Created in BioRender. Wei, X. (2026) https://BioRender.com/sk4j3rj

MASLD arises from dynamic interactions between genetic predisposition and environmental factors, with heritability accounting for approximately 50% of the disease variability [86]. Genome-wide association studies (GWAS) have identified several common genetic variants robustly associated with MASLD susceptibility and progression, all of which encode proteins regulating hepatic lipid metabolism [87]. The I148M variant in *PNPLA3* represents the most significant common genetic determinant; homozygosity for this variant confers up to a 10-fold increased risk of HCC in individuals of European population [88, 89]. This loss-of-function mutation impairs the hydrolysis of triglycerides and retinyl esters, leading to lipid accumulation in hepatocytes and HSCs, thereby promoting liver injury and inhibiting antifibrotic pathways [90, 91]. Additionally, variants in *TM6SF2*, *MBOAT7*, and *GCKR* significantly contribute to MASLD progression [92–94] (Fig. 4a).

The pathogenesis of MASLD is profoundly influenced by dynamic epigenetic modifications that modulate gene expression beyond the DNA sequence itself, creating a molecular interface between genetic predisposition and environmental triggers. These mechanisms critically regulate key aspects of disease progression, including hepatic lipid metabolism, insulin signaling, mitochondrial function, and the oxidative stress response [95]. This multilayered regulatory network encompasses DNA methylation, histone modifications, and non-coding RNAs (ncRNAs). At the level of DNA methylation, promoter hypermethylation of *PPARGC1A* (encoding PGC-1α), a master regulator of mitochondrial biogenesis, has been significantly associated with suppressed mitochondrial function and the development of IR in MASLD patients [96] (Fig. 4b). Concurrently, post-translational histone modifiers play divergent roles: the NAD^+^-dependent deacetylase SIRT1 exerts protective effects by promoting fatty acid oxidation and mitigating inflammation, as evidenced by the finding that its liver-specific deletion exacerbates both steatosis and inflammatory responses [97]. Conversely, hyperglycemia-induced activation of the acetyltransferase p300 promotes DNL by facilitating the acetylation and subsequent activation of the transcription factor ChREBP [98] (Fig. 4c). Notably, HDAC11 has recently been identified as a promising therapeutic target for MASLD. The development of a novel highly selective inhibitor, B6, alleviates hepatic steatosis by inhibiting HDAC11 to activate the AMPKα pathway, thereby reducing de novo lipogenesis and promoting fatty acid oxidation [99].

A vast repertoire of ncRNAs further contributes to epigenetic dysregulation. The downregulation of miR-122, the most abundant microRNA in the liver, is among the most consistently observed alterations in MASLD, leading to the derepression of its lipogenic targets [100]. Additionally, miR-34a has emerged as a central node regulating lipid metabolism and stress responses, with its circulating levels closely correlating with histological disease severity [101]. Hsa-miR-21-5p has been demonstrated to promote the progression from MASH to HCC by inhibiting PPARα, leading to altered lipid metabolism, reduced mitochondrial activity, and activation of oncogenic pathways, ultimately driving hepatocarcinogenesis and correlating with worse patient prognosis [102]. The regulatory landscape is further complicated by competing endogenous RNAs (ceRNAs). For example, circular RNAs such as circ_0046366 and circ_0046367 can function as molecular sponges that sequester miR-34a, thereby attenuating its activity, while the long non-coding RNA MALAT1 modulates inflammatory and metabolic pathways through interactions with transcription factors and other miRNAs [103, 104] (Fig. 4d).

In summary, the pathogenesis of MASLD is collectively orchestrated by genetic predisposition and a multilayered network of epigenetic regulatory mechanisms. This intricate interplay carries profound clinical implications. The detection of circulating ncRNAs released into the bloodstream during disease progression offers a promising avenue for non-invasive diagnosis and dynamic monitoring of MASLD severity. More fundamentally, unlike fixed genetic variants, epigenetic marks are inherently reversible, raising the possibility of therapeutic interventions designed to restore a healthy epigenetic landscape. Harnessing this plasticity represents an underexplored frontier in MASLD therapeutics that could complement conventional metabolic targeting and potentially achieve disease modification at the molecular level.

### Gut-liver axis and microbiota dysbiosis

The gut-liver axis is now recognized as a cornerstone in the pathogenesis of MASLD, with a substantial body of evidence underscoring the profound impact of gut microbial communities on regulating hepatic metabolic processes and inflammatory responses [105] (Fig. 4e). The human gastrointestinal tract harbors a diverse and abundant consortium of microorganisms, including bacteria, fungi, viruses, and archaea, that profoundly influence host metabolic homeostasis [106].

Observational studies have consistently revealed associations between MASLD and altered gut microbiota composition. A consistent finding in MASLD patients is a significant alteration in the composition of this gut microbiota, most notably a reduced overall microbial diversity when compared to healthy individuals [107]. Specific taxonomic shifts often include an increased abundance of Gram-negative bacteria from the Bacteroidetes phylum, a depletion of beneficial genera such as *Prevotella*, and a notable reduction in viral diversity, particularly as the disease advances to more severe stages [108, 109]. Crucially, the progression of MASLD, especially to MASH and significant fibrosis, is strongly linked to a state of marked gut dysbiosis and the expansion of pathobionts [110]. For instance, patients with advanced fibrosis demonstrate a distinct enrichment of pro-inflammatory Proteobacteria and specific species like *Escherichia coli* [111]. Notably, emerging evidence suggests that specific gut microbiome signatures may possess predictive power for future MASLD development, further supporting their clinical relevance as associative biomarkers [112].

The causal mechanisms linking dysbiosis to MASLD pathogenesis have been established through mechanistic studies and interventional experiments. An imbalanced microbiota directly compromises intestinal barrier integrity, leading to increased permeability (“leaky gut”) and enhanced systemic absorption of FFAs and microbial products [113, 114]. This facilitates translocation of potent endotoxins like lipopolysaccharide (LPS) into the portal circulation, where LPS activates Toll-like receptors (e.g., TLR4) on Kupffer cells and hepatocytes, triggering pro-inflammatory cytokine cascades and fostering hepatic insulin resistance [115]. Furthermore, gut microbes critically modulate bile acid synthesis and signaling, exerting distant effects on hepatic glucose and lipid homeostasis through receptors such as FXR and TGR5 [116]. Other gut-derived metabolites, including lactate, ethanol, and trimethylamine N-oxide (TMAO), are systemically elevated in MASLD and contribute to the state of chronic low-grade inflammation and metabolic dysfunction [60]. The causal role of the microbiota has been further corroborated by fecal microbiota transplantation (FMT) experiments, in which transfer of dysbiotic microbiota from MASLD patients to germ-free or antibiotic-treated mice recapitulates key disease phenotypes, including hepatic steatosis and insulin resistance [117].

In summary, while observational studies have identified robust associations between gut microbiota signatures and MASLD severity, mechanistic studies and FMT experiments have established the causal pathways through which dysbiosis drives disease progression. These complementary lines of evidence firmly establish the gut-liver axis as an important pathophysiological mediator and unveil it as a highly promising frontier for novel therapeutic interventions. Harnessing this axis therapeutically, however, will require moving beyond empirical approaches toward mechanism-guided strategies that target specific microbial functions rather than broad compositional changes—a challenge compounded by considerable inter-individual variability in gut microbiota composition. Beyond their therapeutic implications, disease-specific microbial signatures also hold significant potential as non-invasive diagnostic and prognostic biomarkers, and integrating microbiome profiling into clinical algorithms could enable earlier detection of high-risk individuals and more precise disease stratification, thereby facilitating personalized management of MASLD.

### Role of ferroptosis, autophagy and cellular senescence in MASLD progression

The pathogenesis and progression of MASLD are driven by a complex interplay of emerging cellular mechanisms beyond simple lipid accumulation, with ferroptosis, defective autophagy, and cellular senescence representing three pivotal, interconnected pathways.

Ferroptosis is a form of regulated iron-dependent cell death distinct from apoptosis, autophagy, and necrosis [118]. It is driven by the accumulation of lipid peroxides and morphologically characterized by mitochondrial shrinkage, increased membrane density, and reduced cristae, without prominent nuclear alterations [119]. Biochemically, ferroptosis involves elevated levels of ROS, lipid peroxidation, and associated genetic reprogramming [120]. Its regulation intersects with multiple metabolic pathways, highlighting its emerging role in metabolic liver diseases such as MASLD [121]. Accumulating evidence implicates ferroptosis in the pathogenesis and progression of MASLD. For example, Li et al. used RNA-seq analysis to demonstrate that arachidonic acid metabolism promotes ferroptosis in a methionine-choline deficient (MCD) diet-induced MASH mouse model, suggesting its potential as a therapeutic target [122]. Similarly, Tong et al. reported that the ferroptosis inhibitor liproxstatin-1 attenuated hepatocyte apoptosis, pyroptosis, and necroptosis in a MASLD mouse model [123]. Further supporting this, Liu et al. showed that zeaxanthin (ZAF), another ferroptosis inhibitor, exerted antioxidative and anti-inflammatory effects in FFA-treated HepG2 cells [124]. GPX4, which is an antioxidant enzyme, serves as a central gatekeeper of ferroptosis. Its upregulation has been shown to ameliorate metabolic injury, solidifying ferroptosis as a critical link between iron dysregulation, oxidative stress, and inflammatory liver damage [125]. Targeting ferroptosis for MASLD therapy focuses on inhibiting iron accumulation and lipid peroxidation. For instance, iron chelators like deferoxamine and radical-trapping agents like ferrostatin-1 have been shown to attenuate liver damage, inflammation, and fibrosis in MASLD mouse models. Compounds such as attractylodin, metformin, urolithin C, and melatonin also demonstrate efficacy in ameliorating steatosis and ferroptosis markers in high-fat diet-induced mice [126] (Fig. 4f). However, substantial knowledge gaps, including the unidentified key cell type initiating ferroptosis and the unknown critical lipids and downstream pathways, currently hinder therapeutic development. Future research must focus on elucidating precise molecular mechanisms, considering patient heterogeneity for personalized strategies, and identifying reliable biomarkers for clinical diagnosis and monitoring.

Autophagy is a conserved lysosome-dependent degradation process essential for cellular homeostasis, responsible for clearing damaged organelles, protein aggregates, and lipids [127, 128]. It encompasses several forms, including macroautophagy, chaperone-mediated autophagy (CMA), microautophagy, and crinophagy, which differ in their cargo delivery mechanisms to lysosomes (Fig. 4g). Among these, macroautophagy, which entails the sequestration of cytoplasmic material into double-membraned autophagosomes for lysosomal degradation, is the most extensively studied hepatic metabolism [129]. In the liver, macroautophagy regulates lipid homeostasis through lipophagy, the selective autophagic degradation of lipid droplets. Dysregulation of this process is increasingly recognized as a key mechanism in the pathogenesis of MASLD [130]. Autophagy-related proteins can exert opposing, nutrient-state-dependent effects on lipid homeostasis. Under fasting conditions, autophagy degrades the nuclear corepressor NCOR1, thereby enhancing PPARα-mediated transcription of fatty acid oxidation genes [131]. Conversely, under nutrient-sufficient conditions, the autophagy-related kinase ULK1 promotes lipogenesis in an autophagy-independent manner by inhibiting the nuclear shuttling of NCOR1 and enhancing LXRα-driven transcription of SCD1 [132]. This dual regulation fine-tunes hepatic lipid anabolism and catabolism. In MASLD pathogenesis, chronic lipid accumulation perturbs this regulatory network, leading to suppressed autophagic flux [133]. The inhibition occurs at multiple steps: early-stage defects involve decreased expression of core autophagy proteins (e.g., MAP1LC3B, Beclin 1, Atg5, Atg7) and calpain-2-mediated degradation of Atg3 and Atg7 [134]. Late-stage blocks result from impaired autophagosome-lysosome fusion due to altered expression of fusion machinery proteins (e.g., Rubicon, Syntaxin17) and saturated fat-induced lysosomal membrane permeabilization [135, 136]. Furthermore, acute versus chronic lipid exposure differentially modulates key upstream regulators like MTORC1 and AMPK, contributing to the dynamic dysregulation of autophagy during disease progression [137]. Preclinical studies show that pharmacological inducers like rapamycin and carbamazepine restore autophagic flux and ameliorate hepatic steatosis [138]. Hormones, vitamins, sodium-glucose cotransporter 2 (SGLT2) inhibitors and lifestyle interventions such as exercise and intermittent fasting also induce hepatic autophagy and reduce lipid accumulation in models of MASLD [129, 139]. However, translating these findings requires overcoming challenges, including the development of liver-specific delivery systems for autophagy modulators and identifying reliable non-invasive biomarkers to monitor autophagic flux in humans.

Cellular senescence is a state of irreversible cell cycle arrest triggered by various stressors, characterized by distinct morphological and secretory alterations, including the senescence-associated secretory phenotype (SASP) [140]. The risk of developing MASLD and its progressive forms, such as cirrhosis, varies considerably among individuals but increases markedly with age [141]. This age-dependent susceptibility is partly driven by the accumulation of epigenetic modifications that disrupt cellular metabolic homeostasis and promote dysfunction. Over time, this leads to an expanded burden of senescent cells, which lose regenerative capacity and instead secrete pro-inflammatory and profibrotic SASP factors that perpetuate tissue damage and impair repair [142]. In MASLD, chronic liver injury accelerates hepatocyte senescence through mechanisms involving DNA damage, and the extent of senescent cell accumulation strongly correlates with disease severity [143]. Given the pivotal role of senescent cells in the progression of MASLD, “senolytics”, which are therapies designed to selectively clear these cells, have emerged as a novel treatment strategy. For example, the combination of dasatinib and quercetin (D + Q) has been shown in preclinical models to selectively induce apoptosis in senescent cells, thereby ameliorating liver inflammation and fibrosis [144]. Early-phase clinical trials are now underway to explore the safety and efficacy of this approach in patients with idiopathic pulmonary fibrosis [145]. This strategy aims to disrupt the chronic inflammatory cycle mediated by the SASP, offering a fresh therapeutic perspective for reversing liver fibrosis (Fig. 4h). However, the precise molecular pathways linking hepatocyte senescence to MASLD progression remain incompletely elucidated, representing a critical area for future investigation.

In summary, ferroptosis, autophagy, and cellular senescence form a pathogenic network that drives hepatocyte death, metabolic dysfunction, and chronic inflammation in MASLD. They represent a promising new frontier of therapeutic targets, with potential strategies ranging from inhibiting ferroptosis and enhancing autophagic flux to clearing senescent cells, aiming to halt or reverse disease progression.

## Current diagnostic approaches of MASLD

The diagnostic frameworks for MAFLD and MASLD, though conceptually aligned, differ in their specific criteria (Table 1). The diagnosis of MASLD requires a comprehensive, multi-step approach that integrates assessment of cardiometabolic risk factors, systematic exclusion of alternative liver diseases, and accurate staging of disease activity and fibrosis, which reflects the condition’s heterogeneous and progressive nature [146, 147].

**Table 1:** Comparative diagnostic criteria for MAFLD and MASLD [6]

Feature	MAFLD	MASLD
Core Prerequisite	Evidence of hepatic steatosis (by imaging, biomarkers, or histology)	Evidence of hepatic steatosis (by imaging, biomarkers, or histology)
Diagnostic Requirement	Presence of **at least two** metabolic risk abnormalities	Presence of **at least one** of five cardiometabolic risk factors
Specific Criteria	1. **Increased waist circumference** (population-/country-specific thresholds) 2. **Elevated blood pressure** (≥ 130/85 mmHg or on antihypertensive treatment). 3. **Elevated fasting triglycerides** (≥ 1.70 mmol/L or on lipid-lowering treatment) 4. **Reduced HDL cholesterol** (< 1.0 mmol/L [men]; < 1.3 mmol/L [women] or on treatment). 5. **Prediabetes** (fasting glucose 5.6–6.9 mmol/L; 2 h post-load glucose 7.8–11.0 mmol/L; or HbA1c 5.7%–6.4%) 6. **Elevated HOMA-IR score** (≥ 2.5) 7. **Elevated high-sensitivity CRP** (> 2 mg/L)	1. **Body mass index (BMI)** ≥ 25 kg/m^2^ (or ≥ 23 kg/m^2^ in Asians) 2. **Fasting plasma glucose** ≥ 5.6 mmol/L (or on drug treatment for T2D) 3. **Blood pressure** ≥ 130/85 mmHg (or on antihypertensive drug treatment) 4. **Plasma triglycerides** ≥ 1.70 mmol/L (or on drug treatment for hypertriglyceridemia) 5. **Plasma HDL cholesterol** < 1.0 mmol/L (men) and < 1.3 mmol/L (women) (or on drug treatment for this lipid abnormality)
Special Populations	Includes individuals with overweight/obesity or T2D as alternative entry points	**Lean MASLD**: Individuals with normal BMI who meet at least one of the above cardiometabolic criteria fall within the same diagnostic framework

**Fig. 5:**
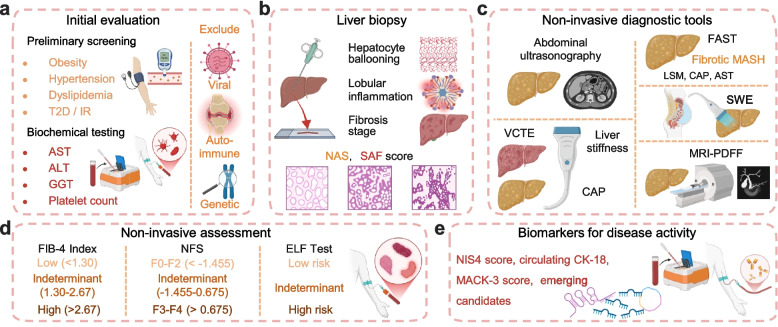
A multi-step diagnostic algorithm for MASLD.** a** Initial clinical and biochemical evaluation. Diagnosis begins with identification of metabolic drivers and signs of hepatic injury. Critically, other causes of liver disease (e.g., viral, autoimmune, genetic) must be rigorously excluded. **b** Liver biopsy remains as the reference standard. Liver biopsy remains indispensable because it provides unique histological information, including hepatocyte ballooning, lobular inflammation, and fibrosis stage. Widely adopted scoring systems, including the NAS and the SAF score, provide a standardized framework for this detailed histological assessment. **c** Non-invasive imaging for steatosis and fibrosis. Abdominal ultrasonography is the first-line imaging modality for detecting steatosis. VCTE simultaneously measures liver stiffness (for fibrosis) and CAP (for steatosis). FAST score, SWE and MRI-PDFF offer alternative approaches. **d** Serum fibrosis biomarkers and risk stratification. The FIB-4 index and NFS are first-line tools to reliably rule out advanced fibrosis. For patients with indeterminate results, second-line tests such as the ELF test provide more precise risk assessment and predict liver-related outcomes. **e** Emerging biomarkers of disease activity. Activity-specific biomarkers are under active investigation. Examples are the NIS4 score, CK-18, the MACK-3 score, and additional candidates, including circulating non-coding RNAs. Abbreviations: T2D, type 2 diabetes; IR, insulin resistance; AST, aspartate aminotransferase; ALT, alanine transaminase; GGT, gamma-glutamyl transferase; NAS, NAFLD Activity Score; SAF, Steatosis, Activity, Fibrosis; VCTE, vibration-controlled transient elastography; CAP, controlled attenuation parameter; FAST, FibroScan-AST; LSM, liver stiffness measurement; SWE, shear wave elastography; MRI-PDFF, magnetic resonance imaging-proton density fat fraction; FIB-4, Fibrosis-4; NFS, NAFLD Fibrosis Score; ELF, Enhanced Liver Fibrosis; NIS4, non-invasive diagnostic test for steatohepatitis 4; CK-18, cytokeratin-18; MACK-3, homeostasis model assessment and aspartate aminotransferase and cytokeratin-18. Created in BioRender. Wei, X. (2026) https://BioRender.com/8u9h4zz

Initial evaluation focuses on identifying metabolic drivers and signs of hepatic injury through clinical and biochemical parameters (Fig. 5a). Key components include screening for features of metabolic dysfunction, such as obesity (assessed by body mass index and waist circumference), hypertension, dyslipidemia, and T2D or IR, in conjunction with liver enzyme profiles [148]. Although serum alanine transaminase (ALT) and aspartate aminotransferase (AST) are widely measured, their sensitivity and specificity for MASLD are limited; an elevated AST/ALT ratio may indicate progressive fibrosis. Additional biomarkers, including gamma-glutamyl transferase (GGT) and platelet count, provide supplementary diagnostic and prognostic information. Critically, other causes of liver disease such as viral, autoimmune, and genetic disorders, must be rigorously excluded [149].

Liver biopsy remains indispensable because it provides unique histological information, including hepatocyte ballooning, lobular inflammation, and fibrosis stage, and it remains the reference standard for diagnosing MASH and enrolling patients in clinical trials [150]. Widely adopted scoring systems, including the NAFLD Activity Score (NAS) and the Steatosis, Activity, Fibrosis (SAF) score, provide a standardized framework for this detailed histological assessment [151, 152] (Fig. 5b). However, the procedure is inherently limited by its invasiveness, associated patient discomfort and rare but serious complications, significant sampling variability due to the heterogeneous nature of liver disease, substantial interobserver disagreement among pathologists, and considerable cost [153, 154]. While serial biopsies remain a common primary endpoint in clinical trials to assess drug efficacy, their impracticality and ethical concerns preclude routine use in clinical care. This stark limitation underscores the urgent and unmet need for robust, validated non-invasive surrogates.

Given the stark limitations of liver biopsy, a growing array of non-invasive diagnostic tools has been developed for MASLD screening and risk stratification. Among these, non-invasive imaging plays a central role in detecting steatosis and estimating fibrosis severity (Fig. 5c). Abdominal ultrasonography remains the first-line imaging modality for identifying hepatic steatosis, though it is operator-dependent and non-quantitative. Vibration-controlled transient elastography (VCTE) enhances diagnostic precision by simultaneously measuring liver stiffness for fibrosis assessment and the controlled attenuation parameter (CAP) for quantitative steatosis evaluation [155]. In clinical practice, VCTE is often employed as a second-line test following initial serum-based screening to confirm fibrosis risk and guide referral decisions [156]. In a prospective MASLD cohort, liver stiffness measurement by VCTE achieved an AUROC of 0.89 for detecting advanced fibrosis [157]. Furthermore, composite scores such as the FibroScan-AST (FAST) score, which integrates liver stiffness measurement (LSM), CAP, and serum AST, have been developed to identify patients with fibrotic MASH. A FAST score ≥ 0.67 indicates high risk for active, fibrotic disease, whereas a score ≤ 0.35 suggests low risk; serial measurements may also reflect histological changes over time [158]. Alternative elastographic techniques include shear wave elastography (SWE) and point SWE (pSWE), the latter of which maintains diagnostic accuracy even in patients with ascites [159]. Magnetic resonance elastography (MRE), based on phase-contrast imaging of shear wave propagation, provides superior quantitative assessment of fibrosis and MASH compared to ultrasound-based methods, with reported AUROCs as high as 0.96 for advanced fibrosis [160, 161]. However, its high cost, limited availability, and requirement for specialized equipment currently restrict its use to specialized academic centers and clinical trials. Magnetic resonance imaging-proton density fat fraction (MRI-PDFF) offers a complementary approach by accurately quantifying hepatic steatosis—enabling diagnosis, grading, and treatment monitoring—though its utility is limited to fat assessment without evaluating necroinflammation or advanced fibrosis, and it is primarily used in research settings or early-phase drug trials rather than routine clinical practice [162, 163].

The non-invasive assessment of liver fibrosis has become a cornerstone in the clinical management of MASLD, enabling widespread risk stratification and guiding subsequent patient management [164]. Serum biomarker panels and clinically derived scores are now widely employed for this initial triage (Fig. 5d). Among these, the Fibrosis-4 (FIB-4) index and the NAFLD Fibrosis Score (NFS) are universally recommended as the first-line tools to reliably rule out advanced fibrosis, particularly in high-risk populations such as individuals with T2D or persistently elevated liver enzymes [146]. The FIB-4 index, which incorporates age, liver enzymes (ALT, AST), and platelet count, efficiently categorizes patients into low (< 1.30), indeterminate (1.30–2.67), or high (> 2.67) risk groups. Due to its excellent negative predictive value, accessibility, and extensive validation, it is regarded as the most clinically useful first-line test [27]. For patients in the indeterminate zone or for more precise risk assessment, proprietary serum panels offer enhanced performance. The Enhanced Liver Fibrosis (ELF) test, which is a serum-based panel measuring hyaluronic acid (HA), procollagen III N-terminal peptide (PIIINP), and tissue inhibitor of metalloproteinase 1 (TIMP-1), assesses fibrosis severity and predicts liver-related outcomes in CLD, though its diagnostic accuracy varies, and often combined with other tests like FIB-4 in screening pathways [165, 166]. Together, these tools provide improved accuracy in detecting significant fibrosis, complementing the existing screening pathways.

Beyond fibrosis staging, emerging biomarkers focused on disease activity (Fig. 5d). The NIS4 score, a non-invasive algorithm combining miR-34a-5p, alpha-2 macroglobulin, YKL-40, and HbA1c, is designed to identify patients with at-risk MASH (NAS ≥ 4 and fibrosis stage ≥ 2) [101]. Circulating fragments of cytokeratin-18 (CK-18), a marker of hepatocyte apoptosis, has shown considerable promise in reflecting the necroinflammatory component of MASH and tracking disease progression [167]. Recently, the MACK-3 score combines CK-18 with the homeostatic model assessment (HOMA) and AST for the assessment of MASH [168]. However, these activity-specific biomarkers remain predominantly research tools, as their validation in diverse populations and standardization across laboratories are still ongoing. Moreover, a growing spectrum of emerging candidates—including glycation markers (AGEs/sRAGE), lipid mediators (eicosanoids), fetuin-A, collagen turnover markers (PRO-C3 and the ADAPT score), fibroblast growth factor 21 (FGF21), and circulating non-coding RNAs—are currently under active investigation, offering potential for improved diagnostic precision [169–171].

Despite significant advances, current non-invasive tests for MASLD face several limitations that prevent them from fully replacing liver biopsy. Serum biomarkers and imaging tools such as FIB-4, the ELF test, and transient elastography demonstrate good utility for excluding advanced fibrosis but exhibit only moderate accuracy for intermediate stages and cannot reliably distinguish simple steatosis from steatohepatitis or quantify inflammatory activity. Consequently, these tools are often employed in combination as an initial screening strategy, with definitive assessment ultimately still requiring liver biopsy. Future directions may lie in integrating multi-omics approaches (genomics, proteomics, metabolomics) with advanced imaging and AI tools to develop composite signatures that reflect disease heterogeneity, enable dynamic monitoring, and ultimately achieve histological-grade accuracy without the risks of invasive sampling.

## Current landscape of MASLD management and treatment

### Lifestyle intervention and metabolic surgery as foundational therapies

**Fig. 6:**
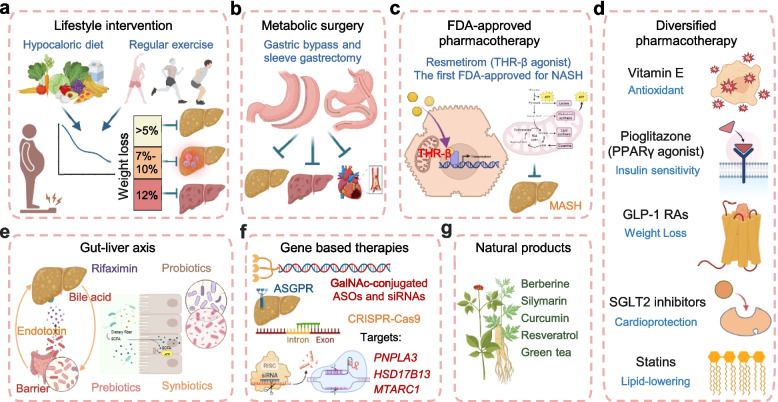
Therapeutic landscape of MASLD. **a** Lifestyle intervention as the cornerstone of management. Clinically significant weight loss achieved through sustained hypocaloric diet and regular physical activity remains the foundational therapy for MASLD. Evidence indicates that weight reduction of ≥ 5% ameliorates steatosis, 7–10% improves inflammatory activity, and ≥ 10% promotes fibrosis regression. **b** Metabolic surgery for severe obesity. For patients with severe obesity in whom lifestyle measures fail to achieve sustained weight loss, metabolic (bariatric) surgery (e.g., Roux-en-Y gastric bypass, sleeve gastrectomy) is highly effective. These procedures induce durable weight loss and reduce the risk of major adverse cardiovascular events and liver-related mortality, even in patients with cirrhosis. **c** Resmetirom: the first approved pharmacotherapy for MASH with advanced fibrosis. Resmetirom, a selective THR-β agonist, acts primarily in the liver to enhance mitochondrial fatty acid oxidation and substrate utilization, thereby improving hepatic lipid metabolism, reducing inflammation, and mitigating fibrosis. **d** Current pharmacological options: approved and repurposed agents. Several established agents provide complementary metabolic benefits, including vitamin E (antioxidant) and pioglitazone (PPARγ agonist), GLP-1 RAs, SGLT2 inhibitors and statins. **e** Targeting the gut-liver axis. Microbiome‑centered interventions (rifaximin, prebiotics, probiotics, synbiotics) aim to restore intestinal barrier integrity, reduce endotoxin translocation, and normalize bile acid metabolism. **f** Gene-based therapies. Nucleotide-based therapeutics, including and GalNAc-conjugated siRNA and ASO, and gene editing technologies like CRISPR-Cas9, enable precise silencing of disease-driving genes such as *PNPLA3*, *HSD17B13*, and *MTARC1*. **g** Natural products and botanical agents. Plant-derived compounds such as berberine, silymarin, curcumin, resveratrol, and green tea catechins exhibit multi-target effects on metabolic, inflammatory, and oxidative stress pathways relevant to MASLD. Abbreviations: THR-β, thyroid hormone receptor-beta; GLP-1 RAs, glucagon-like peptide-1 receptor agonists; SGLT2, sodium-glucose cotransporter-2; siRNA, small interfering RNA; ASO, antisense oligonucleotides; GalNAc, N-acetylgalactosamine. Created in BioRender. Wei, X. (2026) https://BioRender.com/j8mwtl0

Lifestyle modification, centered on clinically significant weight loss achieved through a sustained hypocaloric diet and regular physical activity, constitutes the cornerstone of management for MASLD/MASH [172] (Fig. 6a). Evidence indicates that a weight reduction of at least 5% is required to ameliorate hepatic steatosis, 7%−10% to improve inflammatory activity, and ≥ 10% to promote fibrosis regression [173]. Accordingly, the Asian Pacific Association for the Study of the Liver (APASL) guidelines recommend gradual weight loss (not exceeding 1 kg per week) via a structured hypocaloric diet (creating a daily deficit of 500–1000 kcal) combined with regular exercise—either ≥ 150 min of moderate-intensity or ≥ 75 min of vigorous-intensity activity per week [6]. Lifestyle intervention should be integrated at all stages of care, with the dual objective of improving liver histology and enhancing overall cardiometabolic health. Successful weight loss not only alleviates liver-specific pathology but also confers systemic benefits, including improved glycemic control, blood pressure, lipid profiles, and reduced cardiovascular risk [174].

For patients with severe obesity in whom sustained weight loss through lifestyle modification proves insufficient or unattainable, metabolic (bariatric) surgery offers a highly effective alternative. Common procedures like Roux-en-Y gastric bypass and sleeve gastrectomy induce sustained weight loss and provide significant benefits beyond caloric restriction. These surgeries lead to the resolution of MASH and fibrosis in a substantial proportion of patients, as evidenced by follow-up biopsies [175]. Furthermore, they are associated with a markedly reduced risk of major adverse cardiovascular events and lower long-term overall and liver-related mortality, even in patients with cirrhosis [176] (Fig. 6b). Despite its efficacy, referral rates for eligible patients remain very low, representing a significant gap in current clinical practice.

In summary, lifestyle modification remains the indispensable foundation of MASLD management, with structured weight loss targets directly correlated to histologic improvement. Metabolic surgery offers a highly effective, durable intervention for appropriately selected patients with severe obesity, capable of inducing MASH resolution and reducing long-term mortality. However, the limited reach and poor long-term adherence associated with lifestyle interventions, coupled with the substantial underutilization of metabolic surgery despite its proven efficacy, highlight a critical gap in current care paradigms. These realities underscore the pressing need for accessible, scalable pharmacological options that can replicate or augment the metabolic benefits of these foundational therapies.

### Current pharmacological options: approved and repurposed agents

The demonstrated efficacy of profound metabolic intervention has long underscored the need for pharmacologic strategies that can achieve similar benefits. This need has now been partially addressed by a landmark regulatory approval. Resmetirom represents a paradigm-shifting advancement as the first U.S. FDA-approved pharmacotherapy for MASH, specifically for non-cirrhotic adults with moderate to advanced (stage F2-F3) liver fibrosis. This oral, liver-directed drug is a thyroid hormone receptor-beta (THR-β) agonist that mimics the action of the hormone T3 to improve hepatic lipid metabolism and reduce inflammation and fibrosis. It also enhances mitochondrial fatty acid oxidation and substrate utilization [177, 178] (Fig. 6c). Its accelerated approval in March 2024 was primarily based on the pivotal phase 3 MAESTRO-NASH trial. After 52 weeks of resmetirom treatment, significantly more patients achieved both dual primary endpoints compared to placebo: MASH resolution without fibrosis worsening (25.9% with 80 mg and 29.9% with 100 mg, vs. 9.7% with placebo; *p* < 0.001) and fibrosis improvement by at least one stage without worsening of MASLD activity score (24.2% and 25.9%, respectively, vs. 14.2% with placebo; *p* < 0.001) [179]. Beyond these histological improvements, resmetirom also significantly reduces hepatic fat content, further supporting its metabolic efficacy [180]. While long-term outcomes data are still being collected, this approval ends a decades-long therapeutic drought and establishes a new benchmark against which future agents will be measured.

Moreover, several repurposed agents offer additional therapeutic options for specific patient populations or target distinct pathogenic pathways (Fig. 6d). Among repurposed agents, vitamin E and pioglitazone (a PPARγ agonist) remain the most extensively studied. In the PIVENS trial, vitamin E significantly improved histologic features of MASH compared with placebo (43% vs. 19%, p = 0.001) in non-diabetic adults, whereas pioglitazone, although not meeting the primary endpoint, significantly reduced aminotransferase levels and hepatic steatosis [181]. However, the use of high-dose vitamin E warrants caution, as it has been associated with an increased risk of bleeding, particularly when co-administered with anticoagulant or antiplatelet agents such as warfarin and aspirin [182]. Additionally, data from the SELECT trial suggested a trend toward an increased risk of prostate cancer in healthy men receiving vitamin E supplementation, further tempering enthusiasm for its widespread use in male patients [183].

Glucagon-like peptide-1 receptor agonists (GLP-1 RAs), a drug class originally developed for diabetes and obesity, have demonstrated substantial efficacy in MASLD, underscoring the interconnected nature of metabolic diseases. Meta-analyses of randomized controlled trials confirm that GLP-1 RA treatment significantly reduces liver fat content and improves serum aminotransferases. In patients with biopsy-proven MASH, liraglutide and semaglutide, in particular, are associated with higher rates of histological steatohepatitis resolution without fibrosis worsening [184]. These benefits are mediated primarily through systemic metabolic effects—including weight loss, enhanced insulin sensitivity, and suppressed de novo lipogenesis—rather than direct hepatic action [185]. Consequently, GLP-1 RAs such as semaglutide are increasingly positioned as a first-line pharmacological strategy, particularly for MASLD patients with comorbid obesity or T2D, addressing multiple metabolic abnormalities simultaneously [186].

Similarly, SGLT2 inhibitors (such as empagliflozin and dapagliflozin), another drug class originally developed for diabetes, are increasingly recognized for their metabolic benefits and potential hepatoprotective effects, with overlapping therapeutic targets in patients with MASLD and comorbid T2D [187]. SGLT2 inhibitors exert multifaceted therapeutic effects in MASLD through the activation of hepatocyte autophagy—enhanced via AMPK/mTOR, AMPK/SIRT1, and AMPK/TFEB signaling pathways—which improves lipid metabolism and cellular homeostasis, and concurrently through the promotion of ketogenesis, which facilitates hepatic fat utilization [188]. These integrated actions collectively reduce steatosis, inflammation, and fibrosis, positioning SGLT2 inhibitors as a multi-targeted approach for MASLD management. Notably, emerging evidence suggests that combining GLP-1 RAs and SGLT2 inhibitors may offer synergistic benefits [189].

Statins (HMG-CoA reductase inhibitors) are well-established medications in MASLD management, primarily for managing the elevated atherosclerotic cardiovascular disease risk in this population, where they are considered safe and effective [190]. Emerging evidence suggests potential direct hepatic benefits, particularly in advanced disease. In patients with compensated cirrhosis, statin use is independently associated with preserved global liver function and reduced portal-systemic shunting [191]. These pleiotropic effects, including anti-inflammatory and antioxidant properties, may underlie the observed benefits [192]. Thus, while their core role remains cardiovascular risk reduction, statins show promise as adjunctive therapy for potentially modifying liver disease progression in MASLD.

In summary, the pharmacological landscape for MASLD has evolved from a single reliance on repurposed agents with efficacy but notable safety concerns to a diversified armamentarium anchored by the first approved therapy, resmetirom, for advanced fibrosis. GLP-1 RAs and SGLT2 inhibitors, alongside established drugs like statins, now offer complementary metabolic benefits. The central challenge moving forward lies not in the absence of therapeutic tools, but in their strategic deployment across a highly heterogeneous patient population. Achieving durable, individualized treatment outcomes will require refined strategies for patient stratification—based on disease stage, genetic background, metabolic phenotype, and comorbid conditions—to match the right patient with the right therapy at the right time.

### Emerging therapies in clinical development

Beyond the approved and repurposed agents currently available, a diverse pipeline of investigational therapies is advancing through clinical development, targeting diverse and often complementary pathways involved in MASLD pathogenesis (Table 2). These emerging agents span nuclear receptor modulators, metabolic hormones, and strategies aimed at inflammation and fibrosis, each with distinct mechanisms and stages of evidence.

**Table 2:** Emerging pharmacotherapies in clinical development for MASLD/MASH

Drug Name	Mechanism of Action	Phase	Key Findings/Current Status	Trial number	References
Lanifibranor	Pan-PPAR agonist (α/γ/δ)	Phase 3	Phase 2b showed significant SAF-A score reduction; phase 3 NATiV3 trial ongoing in F2-F3 patients	NCT03008070, NCT04849728	[193]
Saroglitazar	PPARα/γ agonist	Approved in India	Improved ALT, liver fat, insulin resistance, and lipids; authorized for non-cirrhotic MASH in India	NCT03061721	[194, 195]
PXL065	Deuterium-stabilized (R)-pioglitazone, a PPARγ agonist	Phase 2	Improved liver fat, PRO-C3, NFS, and histological parameters including NAS and fibrosis	NCT03967093	[196]
Efruxifermin	Fc-FGF21 analog	Phase 3	Improved hepatic inflammation and fibrosis in F2-F3 patients	NCT04767529, NCT06318169, NCT06215716	[27, 197]
Pegozafermin	Long-acting glycopegylated FGF21 analog	Phase 3	Demonstrated improvement in liver fibrosis in MASH	NCT06318169, NCT06419374	[198]
B1344	Novel FGF21 analog	Phase 1	Study for MASH completed	NCT05655221, NCT07128797	[199]
Aldafermin (NGM282)	Engineered FGF19 analog	Phase 2b	Reduced liver fat, ALT, AST, and fibrogenesis markers; improved NAS and fibrosis scores	NCT02443116, NCT04210245	[200]
Belapectin (GR-MD-02)	Gal-3 inhibitor	Phase 2b/3	No effect on HVPG/fibrosis overall; subgroup analysis in patients without varices showed benefit; NAVIGATE trial (NCT04365868) ongoing for varices prevention	NCT02462967, NCT04365868	[199, 201]
PF-06835919	KHK inhibitor	phase 2	Significantly reduced whole liver fat content and improved inflammatory markers in patients with MASLD	NCT03969719	[202]
Tropifexor (LN452)	Non-bile acid FXR agonist	Phase 2	Sustained improvements in ALT and hepatic fat fraction up to 48 weeks; dose-dependent pruritus	NCT02855164	[203]
Obeticholic Acid (OCA)	FXR Agonist	Phase 3 (Discontinued for MASH)	Showed fibrosis improvement in Phase 3 REGENERATE trial but did not meet criteria for MASH resolution. FDA issued a complete response letter in 2023; development for MASH discontinued	NCT02548351, NCT03439254	[204]
EDP-305	Oral FXR agonist	Phase 2b	Reduced ALT and liver fat at week 12; pruritus as common adverse event	NCT03421431, NCT04378010	[205]
Selonsertib	ASK-1 inhibitor	Phase 3	Phase 3 trials terminated early due to lack of efficacy in F3-F4 fibrosis	NCT03053050, NCT03053063	[206, 207]
Cenicriviroc (CVC)	CCR2/CCR5 antagonist	Phase 3	Reduced fibrosis progression in phase 2b; phase 3 study terminated early due to lack of efficacy	NCT02217475, NCT03028740	[199, 208, 209]
Rifaximin	antibiotic	Phase 4	Significantly reduce circulating endotoxin levels, improve liver enzymes (AST, ALT, GGT), and lower LDL cholesterol and ferritin in patients with MASH	NCT02884037, NCT02009592, NCT01355575	[210, 211]
ALN-MTARC1	a GalNAc-siRNA targeting *MTARC1*	Phase 1	Assessing its safety and tolerability in healthy volunteers and MASLD patients	NCT06265428	[212]
Berberine	an isoquinoline alkaloid	Phase 4	Significantly reduced liver fat, improved IR, and enhanced lipid profiles in patients with MASLD	NCT05647915	[213]
Tirzepatide	GIP/GLP-1 dual agonist	Phase 3	Reduced liver fat by 8.1% over 52 weeks in T2DM patients; improved ALT, AST, GGT	NCT03882970	[214]
Retatrutide	GCGR/GIPR/GLP-1R triple agonist	Phase 3	Significant body weight loss in obese patients; improved liver health markers in preclinical models	NCT04881760, NCT05929066	[199]
VK2809	THR-β agonist	Phase 2b	Reduced hepatic steatosis in mice and liver fat in MASLD patients; phase 2b trial ongoing	NCT02927184, NCT04173065	[199]
MSDC-0602 K	Second-generation Thiazolidinedione (TZD); targets mitochondrial pyruvate carrier	Phase 3	Improved glucose, liver enzymes, and NAS in MASH patients with minimal PPARγ-mediated side effects	NCT03970031	[199]
Aramchol	Stearoyl-CoA desaturase 1 (SCD1) modulator	Phase 3	Phase 2b showed favorable fibrosis and ALT outcomes; phase 3 trial ongoing	NCT02279524, NCT04104321	[199]
Pentoxifylline	Non-specific PDE inhibitor	Phase 3	Improved histological features in phase 2; phase 3 trial ongoing	NCT00590161, NCT05284448	[199]
ZSP1601	Pan-PDE inhibitor	Phase 1b/2a	Improved liver enzymes, fat content, and Fibroscan parameters in MASLD patients	NCT05499208	[215]

PPARs are nuclear receptors that play key regulatory roles in metabolism, inflammation, and fibrogenesis [216]. Similar to pioglitazone, lanifibranor, a pan-PPAR agonist, can improve insulin sensitivity, glycemic control, and lipid profile. In the phase 2b NATIVE trial, lanifibranor resulted in significantly higher rates of MASH resolution without fibrosis worsening and fibrosis improvement without MASH worsening after 24 weeks of treatment. Discontinuation due to adverse events was below 5% across groups. A phase 3 trial (NCT04849728) is currently evaluating the long-term efficacy and safety of lanifibranor over 72 weeks in adults with histologically confirmed MASH and fibrosis stages F2-F3 [193].

FGF21, a hepatocyte-derived hormone central to lipid metabolism, insulin sensitivity, and energy homeostasis, has emerged as a therapeutic target [217]. Long-acting FGF21 analogues, such as efruxifermin, are thus under clinical evaluation for MASH with advanced fibrosis or compensated cirrhosis [198]. In a phase 2b SYMMETRY trial, which enrolled 181 patients with biopsy-confirmed MASH-related compensated cirrhosis, demonstrated that once-weekly 50 mg efruxifermin for 96 weeks led to a significantly higher proportion of patients achieving cirrhosis reversal (defined as a reduction in liver fibrosis of at least one stage without worsening of MASH) compared to placebo (29% vs. 11%) [197]. Based on these findings, the phase 3 SYNCHRONY program has been initiated, comprising SYNCHRONY Histology (NCT06215716) for F2-F3 fibrosis, SYNCHRONY Outcomes (NCT06318169) for compensated cirrhosis, and SYNCHRONY Real-World (NCT06161571) for non-invasively diagnosed disease [27]. These studies aim to definitively establish the long-term safety and efficacy profile of this agent.

Galectin-3 (Gal-3) plays a key role in inflammation and fibrosis in MASLD. The Gal-3 inhibitor belapectin (GR-MD-02) has been investigated as a potential anti-fibrotic therapy. While a phase 2b trial (NCT02462967) in patients with MASH cirrhosis did not meet its primary endpoint for reducing portal pressure overall, a subgroup analysis suggested benefit in patients without esophageal varices [201]. Recently, the phase 2b/3 NAVIGATE trial (NCT04365868), evaluating belapectin for the prevention of esophageal varices in patients with MASH-related cirrhosis, has been completed [199].

Ketohexokinase (KHK) inhibitors are a novel class of investigational drugs targeting pathogenic fructose metabolism for MASLD treatment. They work by inhibiting hepatic de novo lipogenesis, thereby reducing liver fat [218]. Clinical evidence from a phase 2 trial shows that the inhibitor PF-06835919 significantly reduced whole liver fat content and improved inflammatory markers in patients with MASLD [202]. Preclinical studies further support that KHK inhibition ameliorates fructose-induced steatosis and fibrogenesis [219]. Additional candidates, such as GS-1291269, with favorable pharmacokinetics, are in preclinical development [220]. However, research indicates divergent metabolic effects between pharmacological inhibition and genetic knockdown of KHK, highlighting a need for selective inhibitor design [218].

Despite ongoing research efforts, the path to regulatory approval has encountered setbacks that underscore the challenges of translating mechanistic insights into clinical success. Farnesoid X receptor (FXR) agonists, despite their pleiotropic metabolic and anti-fibrotic effects, exemplify this struggle [221, 222]. Tropifexor, for instance, demonstrated dose-dependent reductions in ALT and hepatic fat fraction in a phase II trial, yet its effects on AST were inconsistent and it was associated with dose-related pruritus, a class-wide tolerability issue [203]. Obeticholic acid (OCA), another FXR agonist, advanced to phase III trials but ultimately failed to secure regulatory approval for MASH. Despite demonstrating significant fibrosis improvement with the 25 mg dose, it did not meet the secondary endpoint of MASH resolution, and its development was discontinued amid persistent safety concerns, including a high incidence of pruritus [204]. Similarly, the story of selonsertib, an oral ASK1 inhibitor, highlights the challenges in translational drug development. ASK1 is activated under conditions of oxidative stress and promotes inflammation and fibrosis in MASH via the p38 and JNK signaling pathways [223, 224]. Selonsertib was initially supported by encouraging phase II biomarker data [206]. However, this did not translate into histological efficacy in the phase III STELLAR trials, which showed no significant fibrosis improvement compared to placebo, ultimately leading to the termination of its development for MASH [207]. Targeting inflammatory cell recruitment has also encountered setbacks. Cenicriviroc (CVC), an oral CCR2/CCR5 antagonist, showed initial evidence of fibrosis improvement in the phase 2b CENTAUR trial, but its subsequent phase 3 AURORA study was terminated early due to lack of efficacy [199, 208, 209].

Collectively, these successes and failures illuminate a central truth: MASLD is not a single disease but a heterogeneous syndrome with diverse underlying drivers. The incomplete understanding of its complex pathogenesis, coupled with substantial inter-patient variability in disease presentation and progression, poses fundamental challenges to drug development. No single agent is likely to address all stages and phenotypes of MASLD. The future of effective therapy will therefore depend on refined patient stratification based on genetic background, metabolic phenotype, disease stage, and comorbid conditions, to enable personalized treatment regimens. Rational combination therapies, targeting complementary pathways in parallel, will likely be required to achieve durable histological improvement and, ultimately, to alter the natural history of this multifaceted disease.

## Novel therapeutic targets

### Targeting the gut-liver axis: microbiome-based interventions

Therapeutic strategies aimed at modulating the gut microbiome have emerged as a multifaceted approach to intervening in MASLD progression (Fig. 6e). These interventions primarily work through three core mechanisms: restoring impaired intestinal barrier function, reducing the abundance of endotoxin-producing bacteria, and normalizing dysregulated bile acid metabolism [105].

Rifaximin, a non-absorbable broad-spectrum antibiotic, exemplifies a gut-selective approach. Its efficacy is mechanistically linked to the restoration of the tight junction protein zonula occludens‑1 (ZO1), thereby enhancing gut barrier integrity and reducing the translocation of endotoxins into the portal circulation, as demonstrated in murine models of MASH [225]. Clinical evidences support this mechanism. In a study by Gangarapu et al., short-term administration of rifaximin (1200 mg/day for 28 days) was found to significantly reduce circulating endotoxin levels, improve liver enzymes (AST, ALT, GGT), and lower LDL cholesterol and ferritin in patients with MASH, whereas it demonstrated limited efficacy in patients with simple steatosis [210]. A double-blind, randomized, placebo-controlled trial further reported that 6-month rifaximin therapy was safe and associated with reductions in serum endotoxin, improvements in insulin resistance and proinflammatory cytokines, and decreases in cytokeratin-18 and MASLD-liver fat score in patients with biopsy-proven MASH [211]. These findings indicate that rifaximin may have beneficial effects in select patient populations.

Prebiotics, defined as selectively fermented non-digestible food ingredients that promote beneficial changes in the gut microbiota, have shown preliminary efficacy in MASLD [226]. A small pilot study reported reduced AST levels following supplementation with fructo-oligosaccharides, and a more recent controlled trial demonstrated a significantly greater reduction in hepatic steatosis, as measured by MRI-PDFF, in MASH patients receiving prebiotics compared to the placebo group [227, 228].

Probiotics, which are live microorganisms conferring a health benefit, have been investigated [229]. Specific bacterial strains, particularly multi-strain formulations containing *Lactobacillus*, *Bifidobacterium*, and *Streptococcus*, have been associated with improvements in liver enzymes (ALT, AST), inflammatory markers (e.g., CRP, TNF-α), and serum lipid profiles in several clinical studies [230]. However, the field is hampered by significant heterogeneity in probiotic formulations, dosages, treatment durations, and patient populations, leading to inconsistent results across trials and making definitive conclusions challenging [231].

Synbiotics, which synergistically combine probiotics with their specific prebiotic substrates, offer theoretical benefits for microbial engraftment and persistence [232]. While short-term interventions in metabolically healthy individuals have shown modest reductions in ALT, a rigorous one-year randomized controlled trial conducted in patients with biopsy-confirmed MASLD did not demonstrate significant improvements in hepatic steatosis or fibrosis compared to placebo, highlighting the potential disparity between surrogate markers and hard histological endpoints [233, 234].

Collectively, the current evidence positions gut microbiota modulation as a preliminary area of investigation for MASLD management. While interventions such as rifaximin, prebiotics, probiotics, and synbiotics have demonstrated encouraging effects on surrogate endpoints, their impact on histologically confirmed disease outcomes, particularly fibrosis regression, remains insufficiently established. The field is constrained by substantial heterogeneity in study design, intervention protocols, and patient populations, which limits the generalizability of findings and precludes definitive recommendations. At present, these approaches are best regarded as adjunctive therapies that may complement, rather than replace, established metabolic interventions. By mitigating gut-derived inflammation and enhancing barrier function, they hold potential as components of multimodal treatment strategies.

### Gene-based therapies: from oligonucleotides to gene editing

Nucleotide-based therapeutics, including small interfering RNA (siRNA) and antisense oligonucleotides (ASO), have emerged as a transformative approach in MASLD treatment by enabling the precise silencing of genes implicated in disease pathogenesis (Fig. 6f). This strategy offers a direct route to modulating key pathological phenotypes [235]. A pivotal pharmacokinetic advancement underpinning their clinical application is conjugation with N-acetylgalactosamine (GalNAc), which facilitates highly efficient hepatocyte-specific delivery by targeting the asialoglycoprotein receptor (ASGR) abundantly expressed on liver cells [212].

Carriers of the *PNPLA3* rs738409 (I148M) variant often exhibit hepatic mitochondrial dysfunction that perturbs lipid metabolism and drives MASLD progression [236]. Targeting this key genetic determinant, GalNAc-conjugated ASOs and siRNAs have demonstrated efficacy in preclinical models. Silencing *PNPLA3* in humanized knock-in mice improved steatotic phenotypes, and siRNA specifically targeting the mutant transcript successfully restored a wild-type metabolic profile [237, 238]. Beyond ASO and siRNA, the advent of gene editing technologies like CRISPR-Cas9 holds the potential for a one-time curative treatment for MASLD driven by strong genetic risk factors. For instance, in vitro studies have demonstrated the feasibility of using base editing to introduce the *PNPLA3* I148M mutation in human hepatocyte organoids. Correcting the *PNPLA3* I148M mutation in human hepatocytes through gene editing technologies hold the potential to restore normal triglyceride hydrolysis function [239]. Notably, the *PNPLA3* I148M variant exhibits marked ethnic variability, with the highest allele frequency in Hispanic populations and substantially lower prevalence in individuals of African ancestry [240]. Alongside significant hurdles to clinical translation, including delivery efficiency, off-target effects, long-term safety, and the population-specific nature of such risk alleles, this approach nevertheless represents the frontier of precision medicine for MASLD.

HSD17B13, a lipid droplet-associated protein enriched in hepatocytes, has also been investigated as a therapeutic target [241] (Fig. 6f). In a murine MASH model, *Hsd17b13* ASO administration led to a dose-dependent suppression of its expression and a concomitant reduction in steatosis, although no significant effect on fibrosis was observed [242]. his preclinical finding has been extended to early clinical investigation: subcutaneous delivery of ARO-HSD, an *HSD17B13*-targeting ASO, in healthy volunteers and MASH patients resulted in robust reductions of both mRNA and protein levels, accompanied by decreased serum ALT, indicating improved hepatocyte health [243].

Another emerging target is MTARC1, a mitochondrial outer membrane reductase involved in detoxification [244] (Fig. 6f). Lewis et al. demonstrated that GalNAc-conjugated siRNA targeting *Mtarc1* reduced liver fat and improved certain fibrotic markers in diet-induced MASH mice, albeit without significant changes in overall collagen deposition [245]. Importantly, hepatocyte-specific knockdown of Mtarc1 was shown to improve systemic lipid profiles and attenuate MASH progression in obese murine models [246]. Building on this, a GalNAc-siRNA therapeutic targeting *MTARC1* (ALN-MTARC1) is currently under evaluation in a phase 1 clinical trial, marking an early step toward clinical application [212].

Given that heritability accounts for approximately 50% of MASLD susceptibility, genetic determinants are not merely modifiers but central drivers of disease pathogenesis [86]. This genetic architecture logically positions gene-based therapeutics as uniquely rational interventions capable of addressing the root cause rather than downstream metabolic consequences. The clinical progress of agents targeting *PNPLA3*, *HSD17B13*, and *MTARC1* supports this strategy, demonstrating that precise genetic silencing can translate into measurable improvements in hepatocyte health and metabolic phenotypes. However, it must be acknowledged that this field remains in its infancy. Current evidence is largely derived from preclinical models and early-phase trials, with questions of long-term durability, extrahepatic effects, and delivery efficiency to non-hepatocyte cell populations remaining unresolved. Substantial investment in delivery technologies, safety assessment, and large-scale clinical validation will be essential to determine whether these approaches can be developed into accessible therapies for genetically susceptible MASLD populations.

### Natural products and botanical agents

Botanical medicines and plant-derived extracts represent a rich source of bioactive phytocompounds with documented hepatoprotective properties, offering a complementary and multi-targeted approach to the management of MASLD [247, 248] (Fig. 6g). Among the most extensively investigated natural products are alkaloids (e.g., berberine) and flavonoid complexes (e.g., silymarin), which have demonstrated considerable potential in modulating key metabolic, inflammatory, and oxidative stress pathways central to MASLD pathogenesis.

Berberine, an isoquinoline alkaloid derived from plants such as *Coptidis chinensis*, has garnered significant attention for its pleiotropic benefits, including lipid-lowering, insulin-sensitizing, and broad metabolic regulatory effects [249, 250]. A meta-analysis of six randomized controlled trials reported that at doses of 400–500 mg three times daily for 12–16 weeks, berberine significantly reduced liver fat, improved IR, and enhanced lipid profiles in patients with MASLD [251]. Its efficacy is underpinned by a multi-pronged mechanistic profile: it activates AMPK signaling to inhibit key lipogenic enzymes such as stearoyl-CoA desaturase-1; it suppresses the activation of the NLRP3 inflammasome and subsequent macrophage-mediated inflammation; and it ameliorates hepatic oxidative stress [252–254]. Berberine has been evaluated in a Phase 4 randomized clinical trial (NCT05647915) involving 337 diabetes-free individuals with obesity and MASLD. While 6-month treatment at a daily dose of 1 g did not significantly reduce visceral adipose tissue or liver fat content, it was associated with improvements in LDL-C, apolipoprotein B, and high-sensitivity C-reactive protein levels [213].

Silymarin, a standardized flavonolignan extract from the seeds of *Silybum marianum* (milk thistle) and the active component of the pharmaceutical product Legalon®, has consistently shown hepatoprotective properties in preclinical studies [255]. In an infantile mouse model of MASH, silymarin treatment effectively ameliorated key disease features, including inflammation, apoptosis, hyperglycemia, dyslipidemia, and fibrosis [256]. However, the clinical evidence remains inconclusive. A randomized, double-blinded, placebo-controlled trial in patients with biopsy-proven MASH found that treatment with silymarin 700 mg three times daily for 48 weeks did not meet the primary efficacy endpoint. However, it was associated with significantly greater fibrosis improvement compared to placebo [257]. Combination therapies that pair silymarin (280 mg per capsule) with antioxidants (e.g., vitamins C and E, coenzyme Q10) have shown additive benefits in improving liver biochemistry in MASLD patients, suggesting a synergistic potential [258]. The primary active constituent, silibinin, mediates its protective effects through multiple pathways: it upregulates the Nrf2-mediated antioxidant response, inhibits NF-ĸB-driven pro-inflammatory signaling, activates AMPKα to suppress lipogenesis, and modulates hepatocyte apoptosis via the caspase-8/JNK axis [259–261]. Despite its long history of use, robust and conclusive evidence from large, well-designed clinical trials is still required to confirm its efficacy and define its role in MASLD treatment.

In summary, botanical derivatives such as berberine and silymarin exemplify the therapeutic potential of natural products in MASLD by targeting multiple interconnected pathogenic pathways. Beyond these well-studied compounds, a growing array of other natural agents, including curcumin, resveratrol, green tea catechins, ellagic acid, and *Folium Rhododendri Daurici* extract, have also demonstrated hepatoprotective, metabolic, and anti-inflammatory effects in preclinical and early-phase clinical studies [262–264]. However, despite their historical use and biological plausibility, the clinical evidence for most of these compounds remains limited by small sample sizes, heterogeneous study designs, and a lack of large-scale, rigorously controlled trials. The transition from traditional remedy to evidence-based therapy will therefore require not only further elucidation of their molecular mechanisms but, most critically, validation through standardized, multicenter randomized controlled trials to definitively establish their efficacy, safety, and potential role within future MASLD treatment paradigms.

## Therapeutic challenges and future directions

The pronounced pathophysiological heterogeneity of MASLD, arising from diverse genetic, metabolic, and environmental drivers, remains the central challenge for therapeutic development. Addressing this complexity will require a paradigm shift towards precision medicine, where interventions are tailored to individual patient profiles—their predominant disease drivers, fibrosis stage, and comorbidities. The translation of this vision into clinical reality will depend on a strategic convergence of technologies at different stages of maturity, with some approaches poised for near-term impact while others lay the essential groundwork for a future of personalized care.

In the near term, the most profound impact on clinical practice is expected from the integration of artificial intelligence (AI) into diagnostics and the continued refinement of targeted drug delivery systems. AI-powered digital pathology is rapidly maturing, offering tools to standardize the histological assessment of liver biopsies by automating the quantification of steatosis, inflammation, and fibrosis. This will mitigate inter-observer variability, enhance the precision of clinical trial endpoints, and accelerate the evaluation of new therapies [265]. Simultaneously, AI-driven discovery platforms are already being deployed to mine complex datasets and identify novel drug targets and repurposing opportunities [266].

Complementing this, advanced drug delivery systems are transitioning from academic concepts to clinical reality. The success of GalNAc-conjugated siRNAs (e.g., ALN-MTARC1) exemplifies the power of targeted delivery to hepatocytes, with such agents already in phase 1/2 trials [212]. Similarly, lipid nanoparticles (LNPs) have emerged as versatile, clinically validated nucleic acid delivery platforms. Composed of ionizable lipids, phospholipids, cholesterol, and PEG-lipids, LNPs protect their payload and facilitate cellular uptake and endosomal escape, exemplified by the approval of the LNP-formulated siRNA patisiran [267, 268]. Their utility extends beyond passive hepatic accumulation; functionalization with targeting ligands—such as GalNAc for hepatocyte delivery, vitamin A for activated HSC targeting, or anisamide for extrahepatic applications—enables cell-type-specific intervention across multiple liver pathologies [269].

In the longer term, the ultimate goal of fully personalized medicine will be realized through the integration of human-based disease models, multi-omics-driven patient stratification, and rationally designed combination therapies. Advanced human in vitro models, particularly patient-derived organoids and organs-on-chips, are poised to become indispensable tools in this precision medicine paradigm. Organoids and organs-on-chips (OoCs) recapitulate key structural and functional aspects of human tissues in three-dimensional (3D) formats [270]. These systems enable precise regulation of fluid flow, mechanical cues, and biochemical gradients, thereby establishing microphysiological systems (MPS) that simulate organ-level or even multi-organ (patho)physiology [271, 272]. As human-derived models, both organoids and OoCs overcome the limitations of interspecies variation inherent in animal studies, offering a more physiologically relevant platform for studying human disease. Their application in MASLD research holds significant potential: patient-specific organoids can be used to model individual disease phenotypes and genetic backgrounds, while multi-tissue OoCs—for instance, linking liver and intestinal modules—can replicate the gut-liver axis interactions central to MASLD pathogenesis. These systems allow for high-fidelity simulation of disease processes such as steatosis, inflammation, and fibrosis, facilitating more predictive preclinical evaluation of drug efficacy and toxicity.

The insights gained from these models, combined with systematic integration of multi-omics data (genomics, transcriptomics, proteomics, and metabolomics), will enable the definition of distinct disease endotypes—for instance, identifying patients driven by PNPLA3 risk alleles versus those with predominant gut dysbiosis. This stratification will, in turn, guide the rational design of combination therapies that concurrently target multiple pathogenic pathways. A patient with insulin resistance and advanced fibrosis, for example, may benefit from pairing a GLP-1 receptor agonist for metabolic control with an anti-fibrotic agent. The successful implementation of this vision, however, remains contingent on the parallel validation of robust, non-invasive biomarkers capable of accurately monitoring MASH activity and dynamic fibrotic changes to guide treatment decisions.

In summary, the future of MASLD management may follow a two-pronged trajectory. Near-term advances in AI and targeted delivery are positioned to refine diagnosis and enable cell-specific intervention. These foundational tools may converge with longer-term developments in patient-derived disease models, multi-omics stratification, and rational combination therapy to advance precision medicine: tailoring mechanism-based interventions to individual patients.

## Conclusion

MASLD has evolved from a poorly defined entity to the most prevalent chronic liver condition worldwide, imposing a substantial and escalating burden on global health systems. This review has synthesized the remarkable progress made in understanding its pathogenesis, refining diagnostic approaches, and developing therapeutic strategies, highlighting both the achievements and the formidable challenges that persist.

The pathogenesis of MASLD is a complex, multi-system process originating from a fundamental disruption of energy homeostasis. As detailed throughout this review, metabolic dysregulation (driven by insulin resistance, adipose tissue dysfunction, and aberrant hepatic lipid handling) serves as the primary and essential driver. This initial metabolic insult establishes a state of chronic lipotoxicity, which acts as the critical trigger for a cascade of secondary pathogenic events. As illustrated, lipotoxicity directly compromises mitochondrial integrity and function, leading to oxidative stress and the release of DAMPs. This, in turn, ignites a chronic, low-grade inflammatory response orchestrated by innate and adaptive immune cells, and activates hepatic stellate cells, culminating in progressive fibrogenesis. An integrated view of these pathways reveals a clear temporal and hierarchical relationship: metabolic dysregulation is the foundational initiator; mitochondrial dysfunction and oxidative stress are the key amplifiers; and inflammation and fibrosis are the principal executors of tissue damage and disease progression. Furthermore, the relevance of these pathways is highly stage-dependent. While metabolic drivers are paramount in the early stages of simple steatosis, the inflammatory and fibrotic cascades become increasingly dominant as the disease advances to MASH and cirrhosis, ultimately determining clinical outcomes. Superimposed on this framework are genetic predispositions, dynamic epigenetic modifications, and gut microbial dysbiosis, which modulate an individual’s susceptibility and the rate of disease progression across all stages.

The clinical consequences of this progressive pathophysiology are profound. While cardiovascular disease remains the leading cause of mortality across the MASLD spectrum, the risk of liver-related morbidity and mortality escalates exponentially with advancing fibrosis. Among these, the burden of HCC is of particular concern. MASLD-associated HCC is now a leading cause of liver cancer and can arise even in the absence of cirrhosis, complicating surveillance strategies. The rising global prevalence of MASLD portends a parallel increase in HCC incidence, underscoring the urgent need for effective interventions to halt disease progression before the point of no return.

In parallel with mechanistic insights, the field has witnessed a revolution in non-invasive diagnostics. Tools such as FIB-4, VCTE, and MRI-PDFF now enable widespread risk stratification and have become cornerstones of clinical management, effectively reducing the need for liver biopsy in many patients. However, significant gaps remain. Current non-invasive tests excel at excluding advanced fibrosis but are less accurate for intermediate stages and cannot reliably distinguish simple steatosis from steatohepatitis, nor can they replace biopsy for definitive diagnosis in complex cases or for enrollment in clinical trials. The future of diagnostics lies in the integration of multi-omics biomarkers with advanced imaging and AI to develop composite signatures that reflect disease heterogeneity and dynamic activity with histological-grade accuracy.

The therapeutic landscape for MASLD has been transformed by the landmark FDA approval of resmetirom, the first pharmacotherapy for MASH with advanced fibrosis, ending a decades-long drought. This milestone validates the strategy of direct hepatic targeting and provides a new benchmark for the field. However, it is not a panacea. The growing armamentarium now includes repurposed agents like vitamin E and pioglitazone, as well as metabolic drugs such as GLP-1 receptor agonists and SGLT2 inhibitors, which offer complementary benefits by addressing systemic metabolic dysfunction. Looking ahead, a robust pipeline of investigational therapies, targeting FXR, PPARs, FGF21, and inflammatory pathways, offers further promise, though the field has also learned sobering lessons from late-stage failures (e.g., selonsertib, obeticholic acid), underscoring the critical gap between biomarker signals and histological efficacy.

The central challenge that emerges from this synthesis is the profound heterogeneity of MASLD. It is not a single disease but a syndrome with diverse genetic, metabolic, and environmental drivers, manifesting across a spectrum of histological stages and clinical outcomes. No single agent is likely to address all patients. The future of MASLD management, therefore, lies in a precision medicine paradigm. This will require: (1) refined patient stratification based on genetic background, metabolic phenotype, disease stage, and comorbidities; (2) the rational design of combination therapies that target multiple pathogenic pathways in parallel; and (3) the continued validation of dynamic, non-invasive biomarkers to guide treatment decisions and monitor response. As our understanding of the intricate networks driving MASLD continues to deepen, the integration of these innovative technologies holds the ultimate promise of delivering effective, personalized, and durable interventions for the millions of individuals affected by this global health challenge.

## Data Availability

Not applicable.
